# Season, Irrigation, Leaf Age, and *Escherichia coli* Inoculation Influence the Bacterial Diversity in the Lettuce Phyllosphere

**DOI:** 10.1371/journal.pone.0068642

**Published:** 2013-07-02

**Authors:** Thomas R. Williams, Anne-Laure Moyne, Linda J. Harris, Maria L. Marco

**Affiliations:** 1 Department of Food Science & Technology, University of California, Davis, California, United States of America; 2 Western Center for Food Safety, University of California, Davis, California, United States of America; U. S. Salinity Lab, United States of America

## Abstract

The developmental and temporal succession patterns and disturbance responses of phyllosphere bacterial communities are largely unknown. These factors might influence the capacity of human pathogens to persist in association with those communities on agriculturally-relevant plants. In this study, the phyllosphere microbiota was identified for Romaine lettuce plants grown in the Salinas Valley, CA, USA from four plantings performed over 2 years and including two irrigation methods and inoculations with an attenuated strain of *Escherichia coli* O157:H7. High-throughput DNA pyrosequencing of the V5 to V9 variable regions of bacterial 16S rRNA genes recovered in lettuce leaf washes revealed that the bacterial diversity in the phyllosphere was distinct for each field trial but was also strongly correlated with the season of planting. Firmicutes were generally most abundant in early season (June) plantings and Proteobacteria comprised the majority of bacteria recovered later in the year (August and October). Comparisons within individual field trials showed that bacterial diversity differed between sprinkler (overhead) and drip (surface) irrigated lettuce and increased over time as the plants grew. The microbiota were also distinct between control and *E. coli* O157:H7-inoculated plants and between *E. coli* O157:H7-inoculated plants with and without surviving pathogen cells. The bacterial inhabitants of the phyllosphere therefore appear to be affected by seasonal, irrigation, and biological factors in ways that are relevant for assessments of fresh produce food safety.

## Introduction

The phyllosphere, or total above-ground surfaces of plants, is a habitat for a variety of microorganisms [1]. At 10^5^ to 10^7^ cells/g plant material, bacteria are typically the most abundant colonizers in the phyllosphere and constitute approximately 10^26^ cells globally [2]. Plant pathogens can be members of the microbiota, but the majority of inhabitants are commensal without a known direct detriment or benefit to the plant [1,3]. Species of *Erwinia* and *Pseudomonas* represent some of the most commonly studied plant epiphytes, although recent investigations have shown a much broader diversity of microorganisms in the phyllosphere, including human pathogens and other bacterial species not previously found in that microbial habitat [4–9].

Leafy green produce has been associated with numerous large outbreaks of foodborne illness and is now regarded to be a significant vector of human pathogens [10]. Many of these outbreaks were traced back to the Salinas Valley, CA, USA, where more than 70% of U.S.-produced lettuce is grown. In particular, enterohemorrhagic Escherichia coli, such as serotype O157:H7, has been linked to consumption of leafy greens [11,12]. Field-based studies investigating *E. coli* O157:H7 population dynamics on lettuce found that the pathogen does not typically colonize plants in high levels but can persist in low numbers, on a fraction of plants, long after inoculation [13–16]. However, because the infective dose of *E. coli* O157:H7 is as few as 10 cells, even a low amount of the pathogen is considered a threat for human infection [17,18].

A variety of environmental parameters including ultraviolet light, relative humidity, and temperature likely influence the viability of *E. coli* O157:H7 on plants [19–21]. The indigenous phyllosphere microbiota might also be influenced by these parameters and then contribute (in)directly to the promotion or prevention of *E. coli* O157:H7 survival [20,22,23]. However, the contributions of these microorganisms to the long-term persistence of the pathogen are currently unclear. Investigations into pathogen-microbiota interactions are difficult because although the phyllosphere microbiota have been identified from multiple plant species, geographical locations, and environmental regimes [5,7,24–26], the relative dominance of different (a) biotic factors on the epiphytic bacterial diversity for any single plant species has yet to be investigated. In this study, our aim was to identify and compare the bacterial diversity in the lettuce phyllosphere at a single geographical site over time during four field trials that included different irrigation methods and inoculation with an attenuated strain of *E. coli* O157:H7. This information is needed to systematically assess whether specific field-associated microorganisms or microbial consortia contribute to the differential survival of human pathogens on leafy green produce.

## Materials and Methods

### Field conditions and inoculation of *Escherichia coli* O157:H7

A field in the Salinas Valley, CA, USA was the site of four field trials in 2009 and 2010. Permits and approvals for use of this United States-owned land were granted by the United States Department of Agriculture. Trials conducted in June were designated as early season (E) and August and October as late season (L). Experimental design of the field trials was described previously [16]. Briefly, plants were grown according to standard commercial practices in a split-block design with three blocks irrigated by overhead-sprinkler (sprinkler) and three blocks irrigated by surface-drip (drip). Temperature and relative humidity measurements were recorded at least every 15 min in the field with a HOBO weather station data logger (Onset, Bourne, MA). For each field trial, 4-week old Romaine lettuce cv. Green Towers (*Lactuca sativa*) plants were spray-inoculated in the morning (9-10 am) using spray bottles containing 10^7^ CFU/ml of a rifampicin-resistant isolate of *Escherichia coli* O157:H7 ATCC700728 (*E. coli* O157:H7) (American Type Culture Collection, Manassas, VA USA) in a suspension of 0.1% peptone [16] buffer. Strain ATCC700728 is an attenuated, non-toxigenic, strain of *E. coli* O157:H7 lacking *stx1* and *stx2*, the genes for shiga-like toxin [16]. *E. coli* O157:H7 was delivered by a single spray in an amount of approximately 1 ml to yield a total inoculum density of 10^7^ CFU/plant. In all field trials, control plants were collected from the same block and were not sprayed. In 2010, an additional set of control plants were collected that were sprayed with 0.1% peptone buffer*. E. coli* O157:H7 cell density in the spray bottles was confirmed by enumeration on tryptic soy agar (TSA) containing 50 µg/ml rifampicin.

### Lettuce sampling

Following inoculation of *E. coli* O157:H7, 48 lettuce plants per time point were randomly sampled at 0 and 2 hours post-inoculation (hpi) and then at 2, 7, 14, 21 and 28 days post-inoculation (dpi). Typically 12 sprinkler- and 12 drip-irrigated lettuce plants were collected from each of the inoculated and control treatment groups. Lettuce heads were harvested by cutting approximately 3 cm above the soil surface with a sterile scalpel and then individually packed in sterile 1.6 L Whirl-Pak bags (Nasco, FT. Atkinson, WI). The bagged plants were immediately placed on ice in a cooler for transport to the laboratory for further processing within 24 h after collection.

### Enumeration of bacteria in the phyllosphere

Enumeration of the bacterial cell densities on lettuce was performed for whole lettuce plants collected until 14 dpi. At 21 and 28 dpi, the plants were too large for processing, and therefore all of the outer leaves and leaves located concentrically inwards up to 50 g of plant were collected for analysis. The older, outer leaves were selected because they were present at the time of *E. coli* O157:H7 inoculation. The lettuce was submerged in 0.1% peptone buffer in Whirl-Pak bags (50 to 250 ml) and immediately washed by either homogenization or sonication to dislodge the bacterial cells. For the 2009 field trials, lettuce was homogenized in a Stomacher 400 laboratory blender (Seward, Westbury, NY) for 2 min at medium speed [16]. For the 2010 field trials, the lettuce was sonicated for 7 min in a Branson 8510 Ultrasonicator water bath (Branson Ultrasonics Corporation, Danbury, CT). An automated spiral plater (Autoplate 4000, Spiral Biotech Inc., Boston, MA) was then used to plate serial dilutions of leaf washes onto TSA containing 25 µg/ml natamycin to inhibit fungal growth [27]. Bacterial concentrations were enumerated on TSA after an overnight incubation at ambient temperature (approximately 22°C). The remaining homogenate or sonicate leaf washes were transferred to 50 ml tubes and centrifuged at 3220 x g for 15 min. The supernatant was decanted and the pellet stored at -80 °C until DNA extraction.

Up to and including 7 dpi, culturable *E. coli* O157:H7 ATCC700728 were enumerated from the lettuce washes by plating serial dilutions on TSA containing 50 µg/ml rifampicin. At 14, 21, and 28 dpi, the amounts of the *E. coli* O157:H7 inoculant were too low for detection by plating, and hence enrichment was performed by submerging the plants in approximately 150 ml of tryptic soy broth (TSB) containing 50 µg/ml rifampicin and incubating at 42°C overnight. Cell suspensions were then plated onto CHROMagar O157 (BD, Franklin Lakes, NJ) and incubated at 37°C overnight. The presence of viable *E. coli* O157:H7 cells in the enrichments was indicated by the presence of magenta-colored colonies on CHROMagar.

### DNA extraction

Genomic DNA was extracted from the concentrated cell suspensions washed from plants using a modified protocol employing the QIAamp DNA Stool Mini Kit (Qiagen, Hilden, Germany). Briefly, 180 µl of the frozen leaf wash (between 10 to 25% of the cell pellet) was added to lysis buffer (200 mM NaCl, 100 mM Tris, 20 mM EDTA, and 20 mg/ml lysozyme, pH 8.0) in sterile 2 ml tubes containing 0.5 g of 0.1 mm zirconia/silica beads (Biospec Products, Bartlesville, OK). Samples were shaken for 2 min at 6.5 m/s in a MP FastPrep-24 tissue and cell homogenizer (MP Bio, Santa Ana, CA, USA) and then incubated at 95°C for 5 min to lyse bacterial cells. The resulting genomic DNA was treated with proteinase K and purified on a QIAamp spin column (Qiagen).

### Quantitative real-time PCR

Real-time PCR was used to quantify the total bacterial abundance in the phyllosphere. Universal 16S rRNA primer pairs 534F 5’ CCAGCAGCCGCGGTAAT 3’ and 783R 5’ ACCMGGGTATCTAATCCKG 3’ were used to amplify bacterial DNA while limiting chloroplast DNA amplification [28]. The 20 µl reactions contained 10 µl of SsoFAST^TM^ EvaGreen Supermix (Bio-Rad, Hercules, CA, USA), 2 µl of each primer (12.5 µM stock), and 2 µl template DNA. The reactions were performed on an Applied Biosystems 7500 FAST real-time PCR system (Life Technologies, Foster City, CA, USA) first by heating the samples for 3 min at 95°C and then 40 cycles of 5 s at 95°C and 30 s at 53°C. Bacterial cell densities were estimated using comparisons of Ct values to an *E. coli* O157:H7 genomic DNA standard curve. To confirm the absence of contaminating DNA, negative controls including all reagents except for template DNA were performed for all PCR runs.

### Pyrosequencing and 16S rRNA gene sequence analysis

The 16S rRNA V5 to V9 regions from phyllosphere microbiota genomic DNA were amplified by PCR using bar-coded 799f [29] and 1492r [30] primers as done previously [31]. This primer pair was previously used to amplify 16S rRNA genes from bacteria associated from plants while limiting chloroplast DNA amplification [29]. PCR products were run on an agarose gel and the bacterial DNA band was excised and purified. Negative controls were included in all PCR runs and confirmed the absence of contaminating DNA. The concentrations and qualities of DNA were determined using the Quant-iT PicoGreen double stranded DNA assay (Invitrogen, Carlsbad, CA, USA) and Agilent 2100 bioanalyzer (Agilent, Santa Clara, CA, USA). Equal concentrations of DNA were used for emulsion PCR following protocols developed by Roche 454 Life Sciences. Sequencing was performed at The Core for Applied Genomics and Ecology (CAGE) at the University of Nebraska, Lincoln on the GS-FLX 454 Titanium platform (454 Life Sciences, Brandford, CT, USA). The sequences are available in the NCBI BioProject database with the project identification number SUB148767 (sample accession numbers: SAMNO1882564 to SAMNO1882785).

The Quantitative Insights Into Microbial Ecology (QIIME) [32] software package was used to analyze the 16S rRNA sequences. Prior to taxonomic and phylogenetic analysis, the DNA sequences were subjected to the following preprocessing steps and quality controls: (i) sequences with incorrect bar codes or more than two primer mismatches were removed; (ii) sequences containing windows of 50 consecutive base pairs with an average quality score of less than 20 were truncated at the start of the low quality region; and (iii) sequences with less than 200 bp or more than 600 bp, not including bar codes were also not considered. The data set was de-replicated using cd-hit [33] with a 97% identity cutoff, and labeled as Operational Taxonomic Units (OTUs). Representative sequences for each OTU were aligned in QIIME using the PyNast algorithm [34] and phylogenetic trees of the assigned OTUs were created using FastTree [35]. Chimeras were identified and removed from the data set using ChimeraSlayer [36]. Finally, all OTUs with fewer than 10 reads per sample were removed from the data set.

The QIIME sequence analysis pipeline was used for taxonomic assignment using the Ribosomal Database Project (RDP) classifier and the RDP10 database (training set 6) [37] and for alpha- and beta-diversity sample comparisons according to Phylogenetic Diversity (PD) [38], cluster quality, and the UniFrac distances [39]. Statistical analysis and heat map construction was performed in QIIME and R (http://www.r-project.org). UniFrac distance matrices were created using 300 sub-sampled sequences per sample averaged over 10 iterations and used for principal coordinate analyses (PCoA) and unweighted pair group method with arithmetic mean (UPGMA).

### Identification of *Enterobacteriaceae*

Full-length 16S rRNA sequences of Enterobacteriaceae genera were obtained from RDP and trimmed to the V5-V8 regions (~500 nt) in Bioedit [40]. PyNast was then used to construct multiple alignments of those sequences with OTUs representing unknown Enterobacteriaceae from the phyllosphere. A phylogenetic tree was then constructed using FastTree [35], and the OTUs were either assigned to a specific genus or to a unique clade based on their position in the tree. Sequences assigned to known genera in this analysis were confirmed using the NCBI nucleotide (blastn) database with a minimum 96% sequence similarity cutoff (http://www.blast.ncbi.nlm.nih.gov) and verified by Neighbor Joining in the NCBI blastn software.

## Results

### Bacterial population dynamics in the field

**Figure 1:**
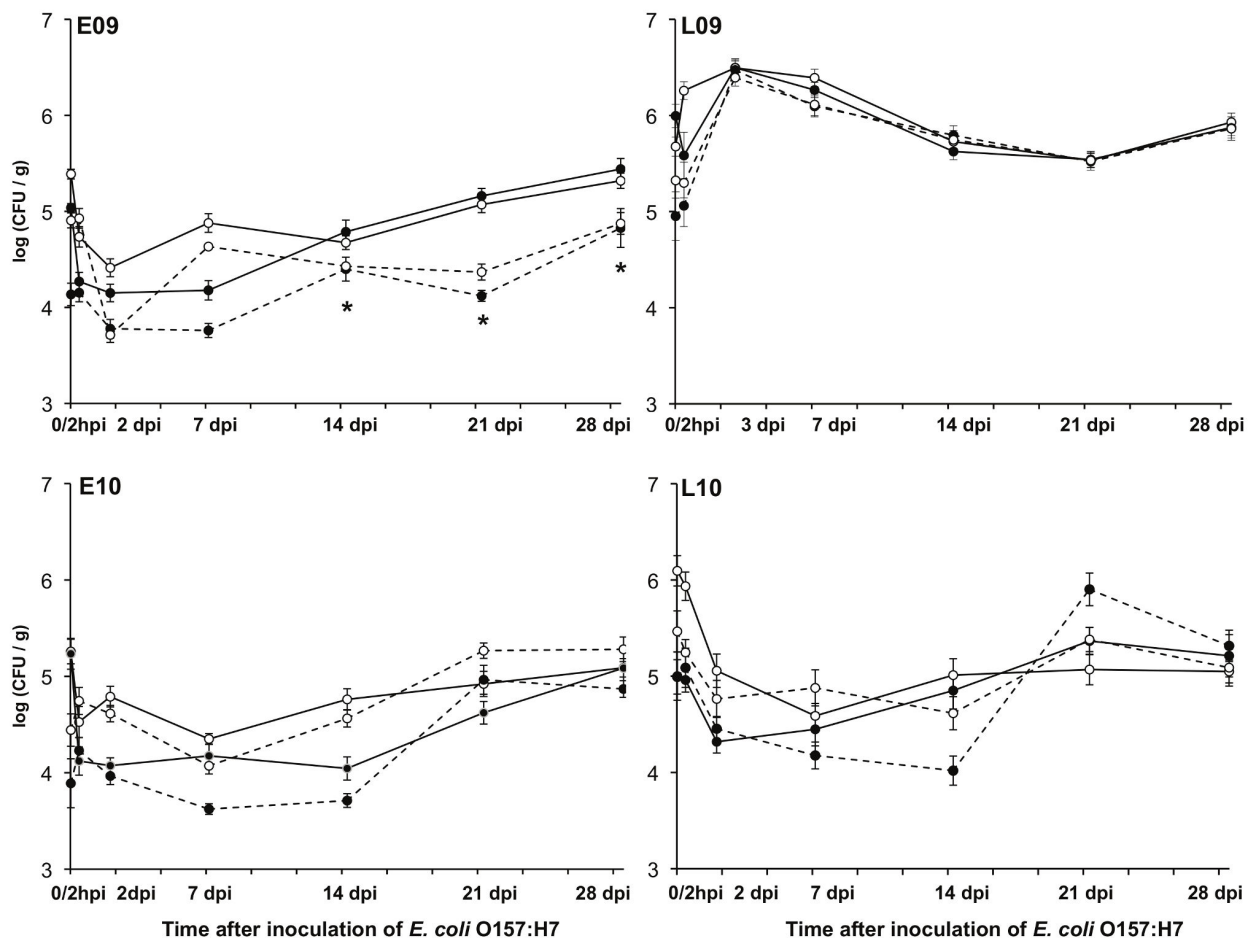
Total culturable aerobic bacterial population sizes on Romaine lettuce during field trials in 2009 and 2010 after inoculation of *E. coli* O157:H7. Culturable bacteria were enumerated on TSA starting at the time of *E. coli* O157:H7 inoculation approximately 1 week after thinning and 4 weeks prior to plant maturity. Overhead irrigated (**○**) and drip irrigated (●) plants were inoculated with *E. coli* O157:H7 (n=12) (solid lines) or non-inoculated controls (dashed lines). Each point represents the average ± the standard error. hpi: hours post inoculation of *E. coli* O157:H7; dpi: days post inoculation of *E. coli* O157:H7; n=12; *n=3 control plants.

In four field trials performed early (E) and late (L) in 2009 and 2010, an attenuated, non-toxigenic strain of *E. coli* O157:H7 was inoculated onto 4-week old Romaine lettuce plants irrigated by sprinkler or drip. *E. coli* O157:H7 persistence and total viable bacterial cell densities were measured until 28 dpi, corresponding to when the plants reached the size typically harvested for human consumption (approximately 8 week-old plants). The number of culturable aerobic bacteria on TSA ranged between log 3.5 and log 6.5 CFU/g plant at all sampling points (Figure 1). However, in general, bacterial abundance in the phyllosphere was lower on plants in the early seasons (E09 and E10) compared to the late (L09 and L10) (Figure 1). Although different lettuce leaf washing approaches were used for years 2009 (homogenization) and 2010 (sonication), we found no evidence that the bacterial abundance was influenced by the recovery method.

The average number of bacteria per gram of leaf tissue increased 3- to 12-fold from 2 dpi to 28 dpi (Figure 1). Except for plants collected in L09, sprinkler irrigated lettuce frequently contained more culturable bacteria than lettuce irrigated by drip (Figure 1). Plants from the L09 trial harbored similar numbers of bacteria independent of irrigation method and the highest bacterial quantities compared with the other trials (Figure 1). The effects observed in L09 might be at least partially attributable to a heavy rainstorm that occurred 2 dpi. Otherwise, the average temperature and relative humidity varied minimally between plantings (Table S1).

Because colony enumerations can underestimate the total number of bacterial cells in the phyllosphere [4,41], we also applied real-time PCR to quantify bacterial abundance on lettuce plants. 16S rDNA-targeted quantification showed that culturing on TSA typically resulted in the detection of only 1 to 10% of the total number of bacterial cells on lettuce (Figure S1). Based on these values, the number of bacteria in the phyllosphere ranged from log 5.1 to log 7.7 cells/g plant (Figure S1). Notably, this estimate is based on real-time PCR using total genomic DNA which might also include DNA from non-viable bacterial cells recovered from the plant surface.

As found previously [16], the number of *E. coli* O157:H7 cells declined rapidly in the lettuce phyllosphere. Within 7 days after inoculation, viable *E. coli* O157:H7 levels were below the detection limit for colony enumeration by plating and only a fraction of the plants were positive (InocPOS) for the organism by enrichment analysis (Table S2). The other *E. coli* O157:H7-inoculated plants lacked any *E. coli* O157:H7 cells (InocNEG), as determined by enrichment.

**Figure 2:**
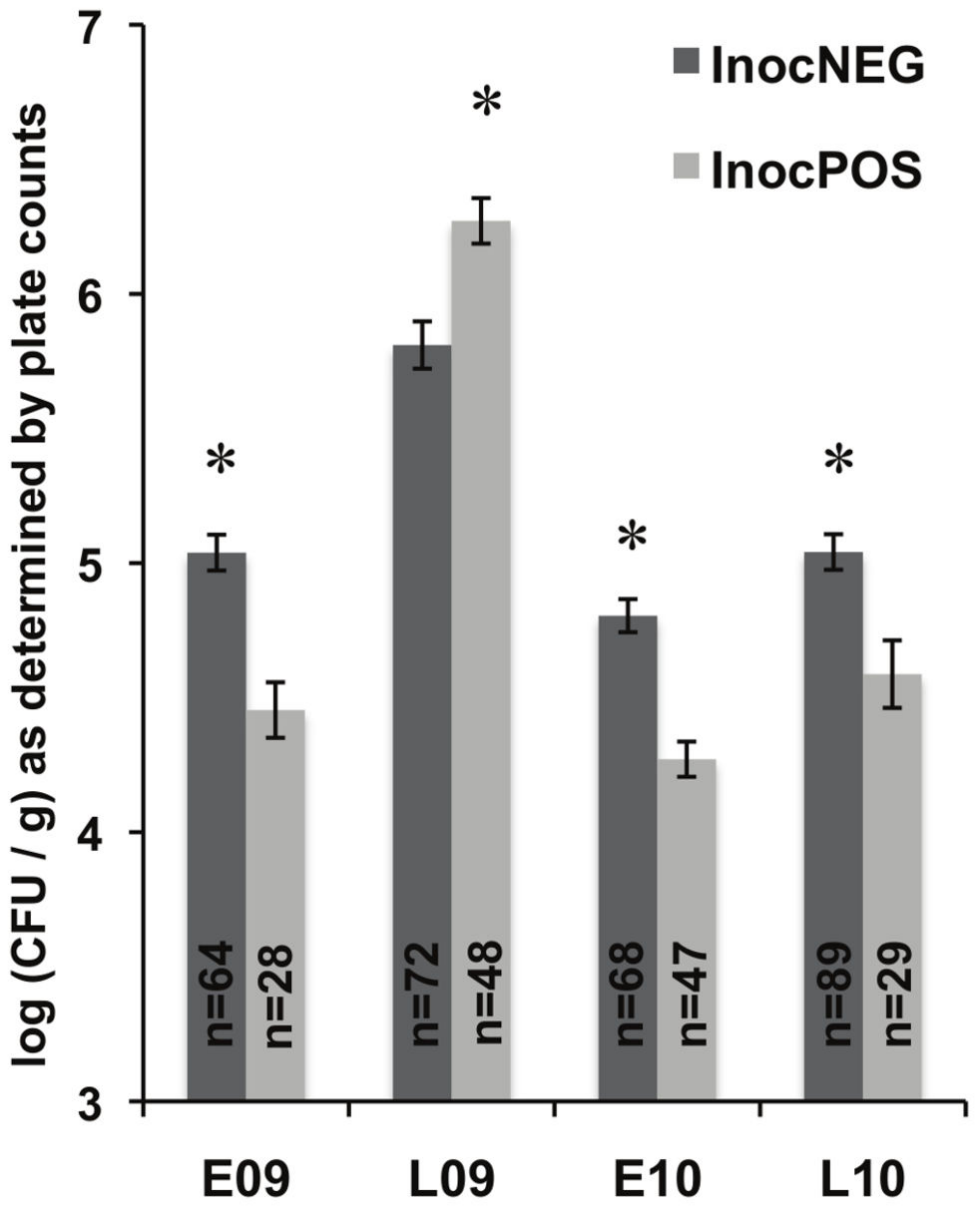
Total culturable aerobic bacterial abundances on plants that were InocPOS or InocNEG for *E. coli* O157:H7. The total aerobic bacterial density enumerated on TSA for inoculated plants with viable E. coli O157:H7 (InocPOS) and those without (InocNEG) averaged for all inoculated plants from 2 to 28 dpi within a field trial. The 0 and 2 hpi time points were omitted due to the presence of large abundance of the E. coli O157:H7; 7 dpi plants were omitted from the E09 trial because enrichment results were not available. An * indicates that significantly more bacteria were found on InocPOS or InocNEG plants by Student’s t-test; P ≤ 0.001.

Season- and irrigation-dependent effects on *E. coli* O157:H7 persistence in the phyllosphere were not found based on comparisons of high numbers of plants in a previous study [16]. However, the influence of the indigenous lettuce surface microbiota was not investigated. Therefore, to identify possible associations between the phyllosphere microbiota and *E. coli* O157:H7 survival on individual lettuce plants, we compared the density of aerobic bacteria cultured on TSA and by real-time PCR for InocPOS and InocNEG plants collected between 2 and 28 dpi. These comparisons showed that lettuce containing viable *E. coli* O157:H7 (InocPOS) harbored significantly lower numbers of bacterial cells compared to the InocNEG plants for the E09, E10, and L10 field trials (Figures 2 and S2). The same result was found for L09 according to bacteria cell enumerations by real-time PCR (Figure S2) but not culture-based assessments (Figure 2).

### Bacterial diversity is limited in the lettuce phyllosphere

We used 454-pyrosequencing to identify the bacteria retrieved from lettuce plants harvested at 7, 14, and 21 dpi from the four field trials (a total of 12 time points). Each time point included treatment groups of sprinkler and drip irrigated plants that were either inoculated with *E. coli* O157:H7 or left as controls. Three uninoculated and six inoculated (three InocNEG; three InocPOS) lettuce plants were typically selected for sequencing for each irrigation treatment at each time point. After quality-filtering of the sequence data, an average of 5,116 sequences per sample remained. The sequences represented a total of 652 OTUs and an average of 36 ± 19 OTUs per sample. Rarefaction analysis confirmed that we sampled the microbiota at a sufficient depth to observe the total bacterial diversity in the phyllosphere (data not shown).

**Figure 3:**
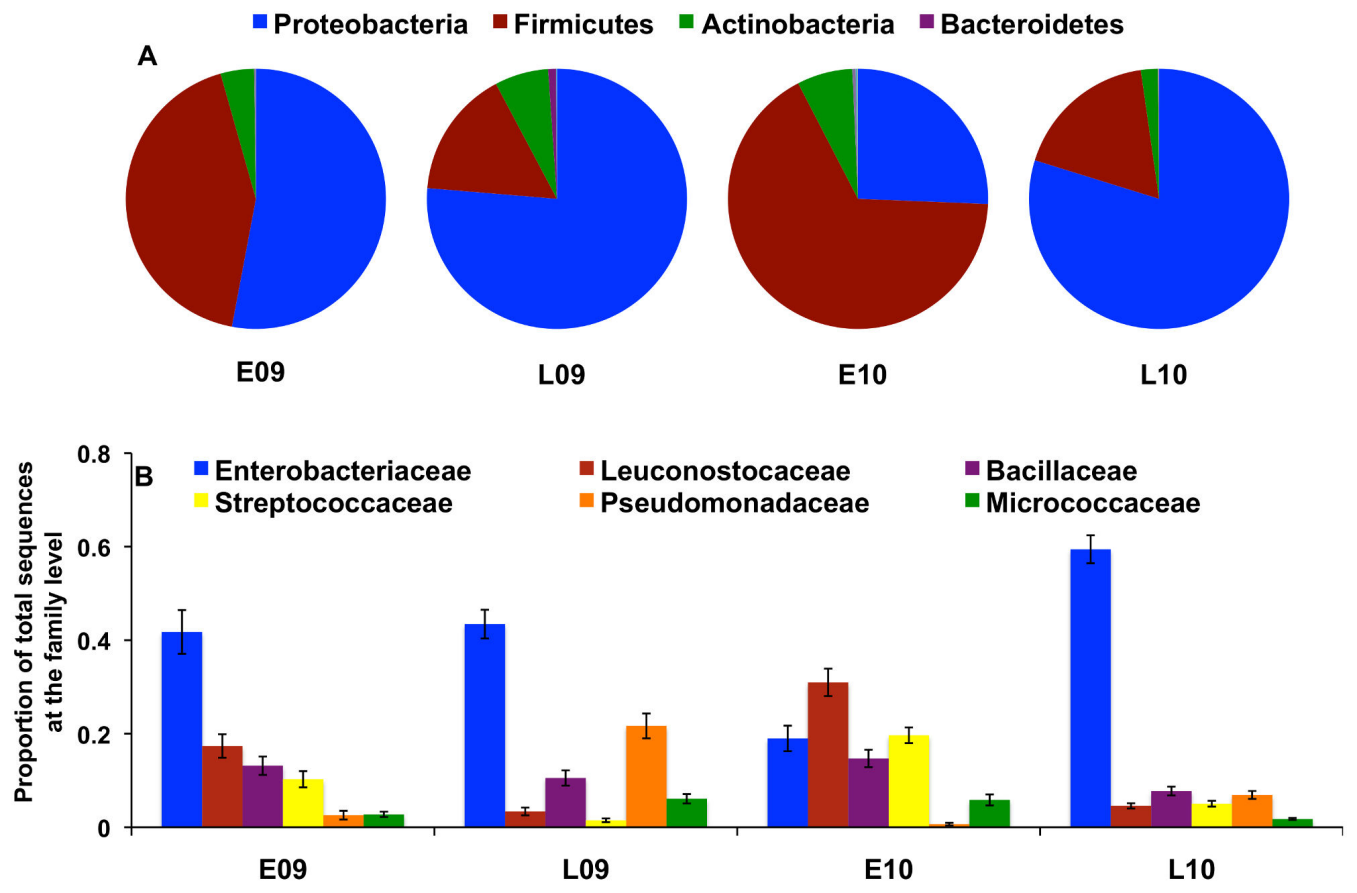
Microbial diversity and seasonal variability in the Romaine lettuce phyllosphere. 16S rRNA sequences obtained by pyrosequencing were quality-filtered and assigned a phylum (A) and family (B) classification. Average relative abundances are shown for each field trial.

Proteobacteria and Firmicutes were the dominant bacterial phyla observed in the lettuce phyllosphere (Figure 3A) and were significantly negatively correlated (R^2^=0.93) (Figure S3). Members of Actinobacteria were also found (Figure 3A), and Bacteroidetes, *Deinococcus* - *Thermus* , Acidobacteria, Gemmatimonadetes, TM7, and *Nitrospira* phyla were present but constituted less than 1% of the total sequences examined.

Nearly all sequences (96%) were classified to at least a bacterial family. Enterobacteriaceae was the most abundant family except for in the E10 planting (Figure 3B). In E10, Leuconostocaceae and Streptococcaceae represented 31% and 20% of the total sequences in that trial, respectively (Figure 3B). Approximately 78% of the sequences could be classified to the genus level for all field trials and the sequences were distributed among 189 genera. Ten genera comprised the majority of bacteria and included *Pantoea* *,* *Leuconostoc* *,* *Pseudomonas* , and *Erwinia* (Figures S4 and S5). In contrast, *Escherichia* was only rarely detected on plants, including plants inoculated with the *E. coli* O157:H7 strain, and when found, was present in amounts totaling less than 0.001% of total sequences.

### Lettuce harbors novel members of the *Enterobacteriaceae* family

The majority of DNA sequences that received a taxonomic assignment only to a bacterial family were members of the Enterobacteriaceae. These sequences, comprising a total of 86 OTUs, were compared to representative sequences from all Enterobacteriaceae genera in RDP. The analysis supported the classification of six OTUs to *Enterobacter* sp., three OTUs to *Erwinia* sp., and single OTUs to *Tatumella* *,* *Citrobacter* *, Raoutella,* *Brenneria* , and *Pantoea* sp. Among the remaining 72 OTUs, 44 were grouped into one of three Clades (Figure S6). Clade 1 contained 17 OTUs and 10% of the total sequences obtained in our study. Clade 2 (19 OTUs) and Clade 3 (seven OTUs) represented 1.4% and 2.0% of all sequences, respectively. Individual OTUs in each clade did not share a common genus-level classification.

### Phyllosphere microbiota varied according to season

**Figure 4:**
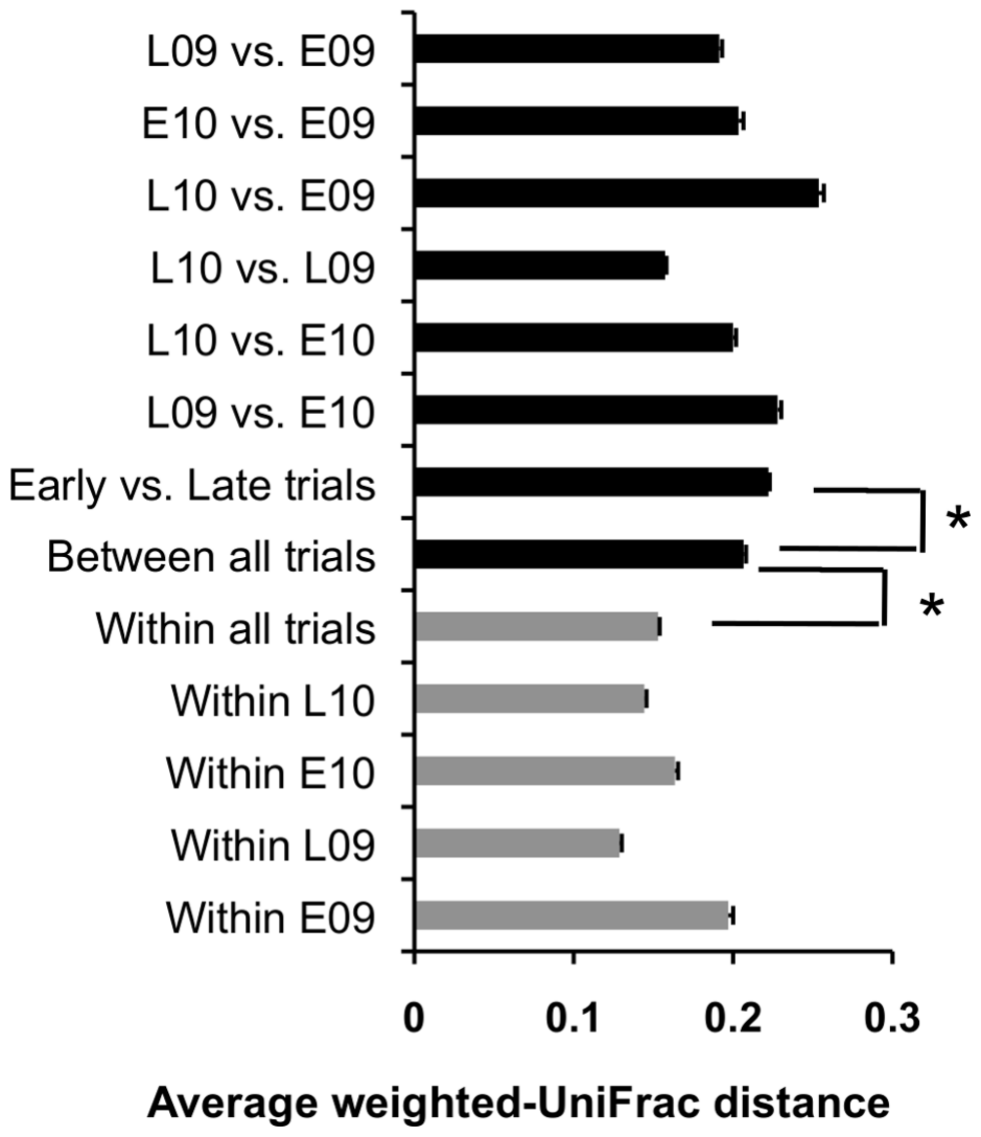
Lettuce phyllosphere community structure between and within field trials. Each bar represents the average and standard error of the weighted-UniFrac distances of pairwise comparisons for each set of samples in the respective subset of data. Black bars represent the average distance between field trials whereas the gray bars are the average distances between samples within trials (* *P* ≤ 0.001 by the Student’s t-test).

Comparisons between the phyllosphere microbiota from lettuce revealed several significant trends. Firstly, the proportions of bacterial genera were distinct on each lettuce plant studied, even among plants collected on the same sampling date (Figure S4). Secondly, pairwise comparisons using weighted UniFrac distances showed that the diversity and proportions of bacteria on lettuce within the same trial were typically more similar to each other than to bacteria collected in other field trials (Figure 4).

**Figure 5:**
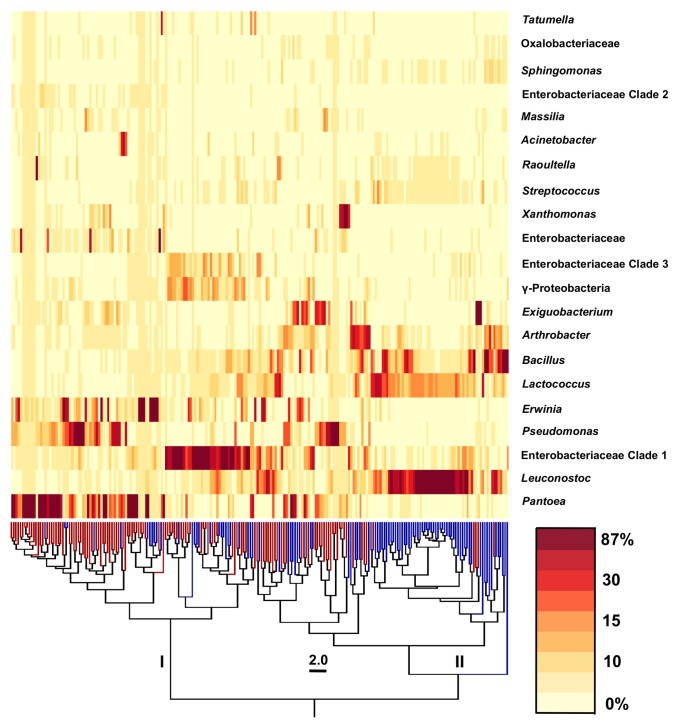
Bacterial diversity phyllosphere differs between season of planting. The UPGMA dendrogram was made in QIIME using weighted UniFrac distance data between phyllosphere microbiota from 223 plants. Tree branches in blue correspond to plants from the E09 and E10 trials and red branches represent samples from the L09 and L10 trials. Clusters I and II were identified as two major sample groups. Relative sequence abundances of the top 21 taxonomic groups are shown in the order of sequence abundance. The heat map indicates a range of proportional sequence abundance in each sample (minimum: 0%; maximum: 87%).

However, despite these differences, there were also similarities between plants and among the field trials. In particular, bacterial diversity on lettuce was associated with the season of planting (early versus late) (Figure 4). To this regard, comparisons between the proportions of abundant bacterial genera on individual plants yielded two clusters (I and II) that were clearly distinguished by the season of planting (Figure 5). Of 109 plants in cluster I, 83 were from the late season (L09 or L10) trials (Figure 5). Proteobacteria were dominant during those field trials and primarily included members of the Enterobacteriaceae and Pseudomonadaceae families (Figure 3A and B). Specifically, *Pantoea* , *Pseudomonas* , *Erwinia* , and Enterobacteriaceae Clade I were the most abundant genera identified in the phyllosphere of late season plants (Figure 5). In cluster II, 74 of 114 plants were from the early season trials. Firmicutes accounted for an average of 32% and 50% of all E09 and E10 phyllosphere bacteria, respectively (Figure 3A). Bacteria retrieved from those plants were predominantly represented by members of the Leuconostocaceae, Bacillaceae, and Streptococcaceae families (Figure 3B) and specifically classified as species of *Leuconostoc* *,* *Lactococcus* *, Bacillus*, and *Exiguobacterium* (Figure 5).

### Temporal and irrigation effects on the phyllosphere microbiota

**Figure 6:**
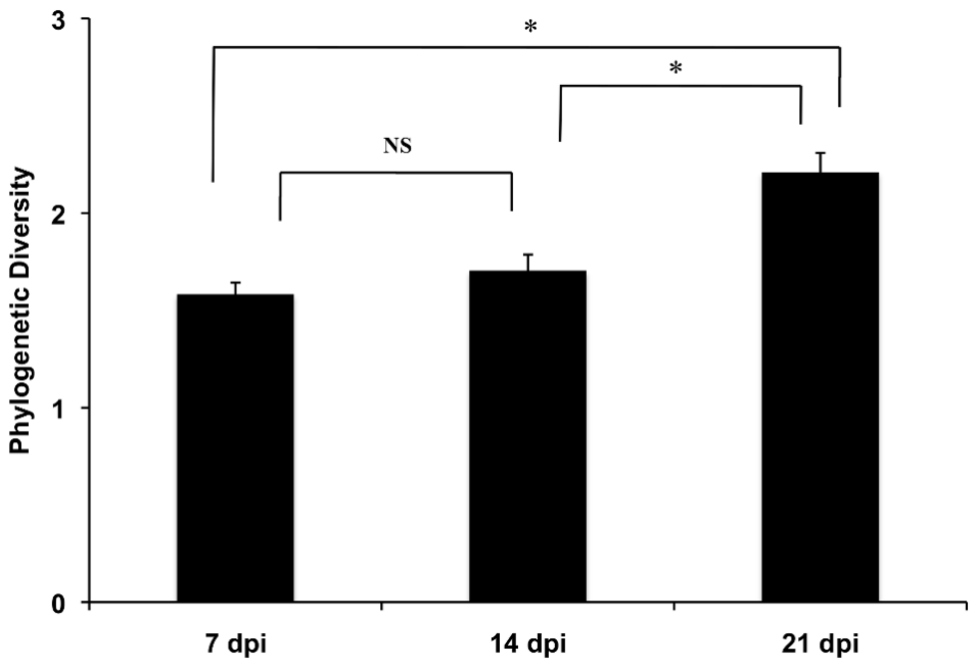
Leaf age influences the diversity of phyllosphere microbial diversity. The Phylogenetic Diversity (PD) of the phyllosphere microbiota from leaves of different ages (dpi). The average ± standard error for all plants the four field trials is shown. n= 73, 84, and 66 for 7, 14, and 21 dpi, respectively; * *P* ≤ 0.001 by the Kruskal-Wallis multiple comparisons test. ns: not significant.

**Figure 7:**
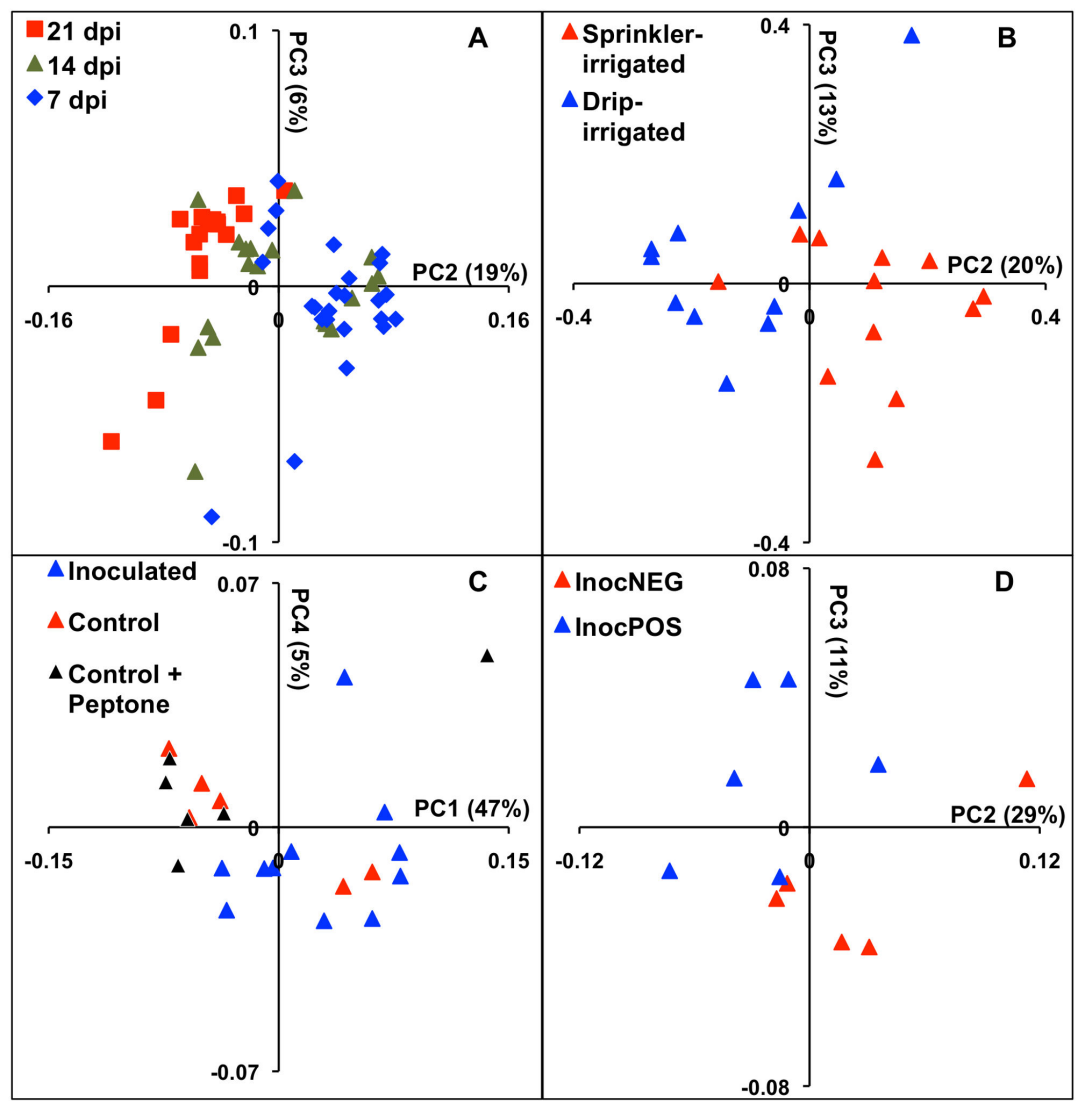
Phyllosphere microbiota differs due to effects of the day of collection, irrigation, and inoculation of *E. coli* O157:H7. Phyllosphere microbiota identified during the L10 were analyzed by Principal Coordinates Analysis (PCoA) performed on Unifrac distances between bacterial communities collected from plants on different days (A), and for plants at 14 dpi exposed to drip or overhead irrigation (B), the inoculation of *E. coli* O157:H7 (C), or inoculated plants that were InocPOS or InocNEG (D). Weighted UniFrac distances were used for (A), (C), and (D) and unweighted UniFrac distances were used for (B). The plots are representative of findings for the other field trials and time points. Additional PCoA plots are in Figure S7.

Within individual field trials, the bacterial composition on lettuce was typically distinct at each time point (Table 1). These differences were at least partially explained by the increased Phylogenetic Diversity (alpha-diversity) on leaves from plants collected at 21 dpi compared to plants at either 7 or 14 dpi (Figure 6). The Chao1 species richness metric also positively correlated with the PD estimator (r^2^=0.89, data not shown). Similarly, temporal changes in the bacterial diversity were identified according to PCoA on Unifrac distances. For example, a temporal change in bacterial composition on lettuce was evident between 7 dpi and 21 dpi in L10 (Figure 7A). At 14 dpi, the microbiota were typically similar to plants collected at either 7 dpi or 21 dpi, possibly indicating a transition from lower to higher bacterial diversity (Figure 7A).

**Table 1:** Cluster quality using weighted UniFrac community analysis.

	**E09**	**L09**	**E10**	**L10**
Day (dpi)	1.129*	1.298*	1.138	1.113*
Irrigation method	1.018	1.057	1.114	1.006
*E. coli* O157 inoculation	1.002	1.004	1.172*	1.031
*E. coli* O157 persistence	0.992	0.967	1.045	1.026

Cluster quality was calculated based on the dissimilarity ratio between/within clusters of samples in a treatment group where a high ratio signifies a distinct cluster. The distance matrices from the weighted UniFrac analysis were used to compute the dissimilarity ratio.

* The highest cluster quality ratio observed in a given planting.

To identify irrigation-dependent effects on the microbiota, it was necessary to compare plants collected on the same day. UniFrac distances examined using ANOSIM revealed that the microbiota on sprinkler- and drip-irrigated plants were significantly different from each other for eight out of the 12 time points sampled (Table 2). The irrigation effects found for one day are shown in Figure 7B. Although no single genus consistently distinguished the bacterial communities between drip and sprinkler irrigated plants, *Xanthomonas* was typically more abundant on sprinkler-irrigated plants and *Erwinia* was enriched on drip-irrigated plants (data not shown). The relative abundance of *Leuconostoc* and *Lactococcus* was also higher on drip-irrigated plants, but only for E09 and E10 plantings.

**Table 2:** ANOSIM cluster analysis of the phyllosphere microbiota between sprinkler- and drip-irrigated Romaine lettuce plants.

	**E09**			**L09**			**E10**			**L10**		
	**7 dpi**	**14 dpi**	**21 dpi**	**7 dpi**	**14 dpi**	**21 dpi**	**7 dpi**	**14 dpi**	**21 dpi**	**7 dpi**	**14 dpi**	**21 dpi**
Unweighted UniFrac	-0.02	-0.006	0.1	**0.13** *	0	**0.55** **	0.22	**0.44** **	0.013	**0.36** ***	**0.33** **	**0.42** **
Weighted UniFrac	-0.16	-0.068	**0.22** *	**0.13** *	0	0.12	**0.67** ***	**0.44** **	0.063	-0.029	0.0026	-0.13

The R-statistic is shown for each comparison of clusters of differentially irrigated plants.

* 0.05 < *P* ≤ 0.1;

** 0.01 < *P* ≤ 0.05;

***P ≤ 0.01

### The phyllosphere microbiota differed on plants inoculated with *Escherichia coli* O157:H7

Inoculation of *E. coli* O157:H7 altered the phyllosphere to the extent that the lettuce microbiota differed between inoculated and control plants for 10 of the 12 time points examined (Figure S7). The result for one of the time points, 14 dpi in L10, is shown in Figure 7C. Notably, the phyllosphere microbiota recovered from naive (not sprayed) and buffer-only inoculated plants were indistinguishable and differences associated with other factors were more important determinants of the bacterial diversity (Figure 7C and S8). Therefore the differences in bacterial inhabitants were most likely due to *E. coli* O157:H7 and not the 0.1% peptone buffer used to deliver the pathogen onto the lettuce.

The bacterial community composition was also distinct between InocPOS and InocNEG plants for nine of the 12 time points sampled, including 14 dpi in L10 (Figure 7D). These differences were partially associated with the enrichment of certain bacteria. For example, at 14 dpi in L10, *Erwinia* was significantly enriched in the phyllosphere of InocNEG plants (*P* ≤ 0.05) according to the Kruskal-Wallis multiple comparison test. *Erwinia* was also enriched on InocNEG plants 2.7-fold and 9.2-fold at 14 and 21 dpi in the L09 trial. However, the opposite trend was seen at 7 dpi in L09, and *Erwinia* was not present at significant levels in E09 and E10.

## Discussion

The bacterial inhabitants of the Romaine lettuce phyllosphere were identified for plants grown in the same field during different times of year over 2 years and exposed to distinct abiotic (irrigation) and biotic (*E. coli* O157:H7) conditions. This work constitutes an in-depth investigation of the microbial community dynamics in the phyllosphere and is the first to examine the possible reciprocal effects between a human pathogen and the indigenous bacterial inhabitants on field-grown plants. We found that the phyllosphere is colonized by a microbiota that is predominantly distinguished by season of planting and shows significant day-to-day variation. Comparisons among plants collected on the same day revealed that the microbiota also differed depending on the irrigation method, inoculation of *E. coli* O157:H7, and the presence or absence of viable *E. coli* O157:H7 cells 7, 14, and 21 days after inoculation.

Overall, the phyllosphere is less diverse compared with other microbial habitats, most notably soil and marine environments [5,8,42–47]. We identified a total of 652 OTUs in the lettuce phyllosphere with each plant containing an average of 36 ± 19 total OTUs. This amount is approximately four-fold less than what was previously reported for Romaine lettuce in the Salinas Valley [31], but comparable to the total bacterial diversity found in the phyllosphere of other plants [5,48]. Moreover, we found that only a limited number of bacteria representing between 1 to 10% of the total cells on lettuce were routinely culturable on standard laboratory medium, and this finding is supported by other investigations of bacterial epiphytes [31,41].

Proteobacteria, Firmicutes, and Actinobacteria were the dominant bacterial phyla identified on Romaine lettuce. These phyla constitute the majority of bacterial inhabitants of numerous plant species [9,31,47–50]. However, unlike other studies, we found higher proportional numbers of Firmicutes. For 67 out of 223 total plants sampled from the field trials, Firmicutes made up greater than 50% of the microbiota and the majority of these plants came from the E09 or E10 plantings. Firmicutes that were represented in high proportions in the phyllosphere included lactic acid bacteria (LAB), and the LAB genera *Leuconostoc* and *Lactococcus* were among the six most abundant genera identified in this study. Other LAB were also found in the phyllosphere in lower abundance, including *Enterococcus* , *Streptococcus* , and *Lactobacillus* . LAB were previously found on lettuce [51], however, these bacteria have generally not been found on plant surfaces in measurable quantities in other studies [31,52]. Such differences might be the result of variation in leaf washing or DNA extraction methods. The latter is likely most critical because we found that different methods for bacterial removal (sonication and homogenization) resulted in the recovery of similar quantities (Figure 1) and diversity of bacteria (Figures 3 and 4). In contrast, DNA extraction methods strongly influenced the diversity of bacteria detected from other habitats [53,54]. In particular, the use of bead-beating improved the extraction of DNA from a wider-variety of bacterial cells, most notably from Gram positive bacteria [55,56]. Clearly, technical differences are important to consider when comparing datasets from multiple investigators, and ultimately efforts should be made to improve protocols so that all bacterial species inhabiting the phyllosphere can be adequately examined.

*Pantoea* and *Erwinia* , among other members of the Enterobacteriaceae, are often common colonists of plants [8,31,47,51,52] and were among the most abundant genera found on lettuce during the field trials. Additionally, we identified three groups of sequences belonging to the Enterobacteriaceae family that were not classified as known genera. Clades 1 and 3 were relatively abundant on both early and late season plants and often found together in the phyllosphere. Clade 2 was found primarily on late season plants. Sequencing errors can lead to the false discovery of unknown OTUs [57], however, because representatives of these clades were found in high amounts on numerous plants from different field trials, the clades we identified might indicate the presence of novel bacterial species unique to the phyllosphere. This finding needs to be confirmed using independent methods designed to detect, quantify, and isolate these bacteria from plants.

The culturable and total cell densities on lettuce were similar for all field trials indicating that the bacterial population sizes on plants are constrained by the carrying capacity of the leaves. In contrast, the relative proportions of bacterial genera inhabiting the lettuce were distinct for individual plants, among plants within a field trial, and also in different field trials. Although the reasons for these differences are not yet known, a number of factors likely influence the diversity of bacteria in the phyllosphere including colonization patterns as plants emerge from the soil, insects, or weather conditions.

Remarkably, even with the significant inter-plant variation among bacteria on lettuce, the microbiota could be distinguished on the basis of the time of year when the plants were collected. This finding is the first time seasonal effects on the diversity of phyllosphere microbiota have been characterized for an annual plant grown in a single location. Phyllosphere communities have previously been shown to differ according to season on cottonwood ( *Populus* *deltoids* ) and magnolia ( *Magnolia* *grandiflora* ) trees [58,59] as well as for bacterial communities in soil [60,61] and air [62]. Seasonal effects were also proposed for the phyllosphere of Romaine lettuce, yet because different geographical locations (California and Arizona) were surveyed, it was not possible to ascertain whether these were season or location-dependent differences [31]. Although there are likely geographical effects on the phyllosphere microbiota [24,63], our results underscore the natural variation in the microbial composition on plants at one site within and between plantings.

Another factor common among the field trials was the increase in diversity and change in composition of the bacteria on lettuce between 7 to 21 dpi. Microbial succession has been reported to occur in the phyllosphere [59,64,65], however, to fully address questions of microbial succession compared with daily and seasonal variations, future studies should aim at studying the bacterial diversity on plants collected at different stages of maturity on the same day in a single geographical location.

Upon limiting our comparisons to plants collected on the same date in each field trial, differences were found between the microbiota exposed to drip or sprinkler irrigation. Colony assessments indicated that there were typically more bacterial cells on plants that were sprinkler-irrigated possibly due to the higher availability of free water on those plants. Similarly, the microbial diversity was also influenced by irrigation method for the majority of plant sampling dates. Although bacteria in the water or in soil-splash might have also contributed to the alteration of the microbiota on the sprinkler-irrigated plants, this possibility is not supported by prior work which concluded that the source of irrigation water was not significantly linked to the microbial diversity in the phyllosphere [8] and the lack of evidence for an increased presence of typical soil-associated bacteria among our sequencing results on sprinkler-irrigated plants.

The inoculation of *E. coli* O157:H7 also resulted in minor, but lasting effects on the composition of the lettuce microbiota. Because few studies have aimed to investigate the effects of a bacterial inoculant on existing bacterial populations on plants [66], it is currently not known whether the observed differences in the bacterial diversity were due specifically to the strain of *E. coli* O157:H7 used in this study or the result of a more general effect that might also apply to other inoculants such as biocontrol agents.

Most notably, microbial community differences were found when inoculated plants were grouped according to the presence/absence of viable *E. coli* O157:H7 cells (InocPOS/InocNEG). Bacterial abundance measured by enumeration on TSA and by real-time PCR indicated that, on average, the total number of cells was lower on plants that contained very low but persistent quantities of viable *E. coli* O157:H7. This finding indicates that under pre-harvest conditions, plants harboring reduced numbers of epiphytes might be more likely to support contaminants such as human pathogens. However, the total number of cells likely only partially explains the differences between plants with and without persistent *E. coli* O157:H7. PCoA plots indicated that the bacterial diversity among epiphytic populations was distinct between InocNEG and InocPOS plants. Thus, it appears that certain bacterial species or microbial consortia in the phyllosphere might contribute to the differential persistence of *E. coli* O157:H7 on plants in the field. This result is in agreement with previous studies examining *E. coli* O157:H7 interactions with individual bacterial strains on growth-chamber grown lettuce [22,67]. Although the specific organisms responsible for these effects in the field are not yet known, we found evidence that the presence of *Erwinia* might be correlated with a decrease in the ability of *E. coli* O157:H7 to survive on plants. *Erwinia* , like *E. coli*, is a member of the Enterobacteriaceae and, as shown previously for other closely related epiphytes [23], these bacteria might compete with the pathogen for the nutritional resources available on the leaf surface. However, because of the significant variability in the composition of the phyllosphere microbiota, it remains to be determined whether species of *Erwinia* can directly influence *E. coli* O157:H7 survival.

We applied high-throughput DNA sequencing to identify and characterize the bacteria on lettuce exposed to biotic and abiotic treatments in a single field over time. The approach is aligned with large-scale projects such as the Earth Microbiome Project (http://www.earthmicrobiome.org) that aim to identify and compare microorganisms in the environment with extensive metadata [68]. The increasing number of outbreaks of foodborne illness due to human pathogen contamination of leafy-green produce has heightened the need for such studies for the specific purpose of improving microbial food safety. Additionally, fundamental information was gained on how bacterial communities develop and the resiliency of those communities against disturbance. Our findings showed that *E. coli* O157:H7 altered the microbial communities on plants, and conversely, the microbiota were distinct on most plants that harbored viable *E. coli* O157:H7 cells long after inoculation. The results of this work might eventually be useful in developing risk-assessment tools to predict pathogen contamination and for new control strategies designed to improve microbial food safety and defense.

## Supporting Information

Figure S1 Culturing on TSA medium underestimates the total bacterial abundance in the phyllosphere. The estimated number of bacterial cells per gram lettuce determined by quantitative real-time PCR targeting the bacterial 16S rRNA genes compared with colony forming units enumerated on TSA. The lines show the percent of total bacteria detected by TSA compared with real-time PCR. Each point represents an individual plant collected at the 7, 14, and 21 dpi time points from each field trial; n=115.
(TIF)

Figure S2 Comparison of bacterial abundances on InocPOS and InocNEG plants as determined by RT-PCR. Total 16S rRNA genes were enumerated by quantitative real-time PCR from a fraction of InocPOS and InocNEG plants and averaged across plants within a field trial from 2 to 28 dpi. * Indicates that significantly more bacteria were found on InocNEG plants with a *P* ≤ 0.10 by the Student’s t-test.
(TIF)

Figure S3 Correlation between Proteobacteria and Firmicutes abundances in the Romaine lettuce phyllosphere. The phyla are inversely correlated with an R^2^ of 0.93. A total of 223 plants were examined during E09, L09, E10, and L10.
(TIF)

Figure S4 Microbial diversity at the genus level in the Romaine lettuce phyllosphere. The top 20 most abundant taxonomic groups at the genus level are listed. The relative abundance for each of the 20 groups is shown for each plant, listed from 7 dpi (left) to 21 dpi (right) for the four field trials. When sequences were unable to be classified to a genus, the next most specific designation is shown (i.e. Family or Class level).
(TIF)

Figure S5 Rank abundance of the top 20 taxonomic groups at the genus level. The ranked average percentage of the most abundant taxa across all samples (n=223) is shown. The samples are ranked in order of abundance from left to right on the x-axis. When sequences were unable to be classified to a genus, the next most specific designation is shown (i.e. Family or Class level).
(TIF)

Figure S6 Classification of unknown 16S rRNA sequences from the Enterobacteriaceae family. The similarity of 16S rRNA sequences from OTU’s classified as belonging to unknown genera of Enterobacteriaceae (numbers) and representative Enterobacteriaceae genera (Genus_species) is investigated here. Those OTU’s that grouped together and not closely related to known genera were designated as belonging to one of three Clades. Individual branches for OTUs within a clade were removed to improve visualization. The average of sequence lengths for the Enterobacteriaceae unknowns (417 bp) was not significantly different from the average read length of all OTUs (431 bp) in the data set (*P* > 0.15, by Student’s T-test). An OTU classified as *Bacillus* was used as an outgroup.
(TIF)

Figure S7 PCoA of UniFrac community distances between *E. coli* O157:H7 inoculated and control plants. Differences between inoculated and control plants are highlighted at each timepoint in all four trials. Unweighted UniFrac community distance data was used to make the E09 14 dpi drip-irrigated PCoA, the remaining analyses used the weighted UniFrac data. All points represent a single microbiota isolated from lettuce. (A) E09; (B) L09; (C) E10; (D) L10.
(TIF)

Figure S8 Comparison of microbiota on control plants examined in 2010. PCoA plots of weighted UniFrac community distances of control plants in the L10 trial. Plant samples are colored according to days post inoculation (dpi) (A) and control group type (unsprayed controls (control) vs. peptone sprayed controls (control + peptone) (B).
(TIF)

Table S1 Temperature and humidity measurements in the field.
(DOC)

Table S2 Percentage of inoculated plants with persistent *E. coli* O157:H7 populations.
(DOC)

## References

[1] Lindow SE, Brandl MT (2003) Microbiology of the phyllosphere. Appl Environ Microbiol 69: 1875-1883. doi:10.1128/AEM.69.4.1875-1883.2003. PubMed: 12676659. 12676659 10.1128/AEM.69.4.1875-1883.2003 PMC154815

[2] Morris CEK, Kinkel LL (2002) Fifty years of phyllosphere microbiology: significant contributions to research in related fields. In: Lindow SE Hecht-Poinar EI Elliott VJ Phyllosphere Microbiology. St. Paul, Minn: APS Press pp. 365-375.

[3] Hirano SS, Upper CD (2000) Bacteria in the leaf ecosystem with emphasis on Pseudomonas syringae-a pathogen, ice nucleus, and epiphyte. Microbiol Mol Biol Rev 64: 624-653. doi:10.1128/MMBR.64.3.624-653.2000. PubMed: 10974129. 10974129 10.1128/mmbr.64.3.624-653.2000 PMC99007

[4] Yang CH, Crowley DE, Borneman J, Keen NT (2001) Microbial phyllosphere populations are more complex than previously realized. Proc Natl Acad Sci U S A 98: 3889-3894. doi:10.1073/pnas.051633898. PubMed: 11274410. 11274410 10.1073/pnas.051633898 PMC31148

[5] Delmotte N, Knief C, Chaffron S, Innerebner G, Roschitzki B et al. (2009) Community proteogenomics reveals insights into the physiology of phyllosphere bacteria. Proc Natl Acad Sci U S A 106: 16428-16433. doi:10.1073/pnas.0905240106. PubMed: 19805315. 19805315 10.1073/pnas.0905240106 PMC2738620

[6] Mark Ibekwe A, Grieve CM, Papiernik SK, Yang CH (2009) Persistence of *Escherichia coli* O157:H7 on the rhizosphere and phyllosphere of lettuce. Lett Appl Microbiol 49: 784-790. doi:10.1111/j.1472-765X.2009.02745.x. PubMed: 19843205. 19843205 10.1111/j.1472-765X.2009.02745.x

[7] Kadivar H, Stapleton AE (2003) Ultraviolet radiation alters maize phyllosphere bacterial diversity. Microb Ecol 45: 353-361. doi:10.1007/s00248-002-1065-5. PubMed: 12704563. 12704563 10.1007/s00248-002-1065-5

[8] Telias A, White JR, Pahl DM, Ottesen AR, Walsh CS (2011) Bacterial community diversity and variation in spray water sources and the tomato fruit surface. BMC Microbiol 11: 81. doi:10.1186/1471-2180-11-81. PubMed: 21510867. 21510867 10.1186/1471-2180-11-81 PMC3108269

[9] Knief C, Delmotte N, Chaffron S, Stark M, Innerebner G et al. (2012) Metaproteogenomic analysis of microbial communities in the phyllosphere and rhizosphere of rice. ISME J 6: 1378-1390. doi:10.1038/ismej.2011.192. PubMed: 22189496. 22189496 10.1038/ismej.2011.192 PMC3379629

[10] Doyle MP, Erickson MC (2008) Summer meeting 2007 - the problems with fresh produce: an overview. J Appl Microbiol 105: 317-330. doi:10.1111/j.1365-2672.2008.03746.x. PubMed: 18284485. 18284485 10.1111/j.1365-2672.2008.03746.x

[11] Center for Disease Control and Prevention (2010) Investigation update: multistate outbreak of human *E. coli* O145 infections linked to shredded romaine lettuce from a single processing facility (final update). http://www.cdc.gov/ecoli/2011/ecoliO157/romainelettuce/032312/index.html. Accessed: June 15, 2012.

[12] Buchholz U, Bernard H, Werber D, Böhmer MM, Remschmidt C et al. (2011) German outbreak of *Escherichia coli* O104:H4 associated with sprouts. N Engl J Med 365: 1763-1770. doi:10.1056/NEJMoa1106482. PubMed: 22029753. 22029753 10.1056/NEJMoa1106482

[13] Barker-Reid F, Harapas D, Engleitner S, Kreidl S, Holmes R et al. (2009) Persistence of *Escherichia coli* on injured iceberg lettuce in the field, overhead irrigated with contaminated water. J Food Prot 72: 458-464. PubMed: 19343931. 19343931 10.4315/0362-028x-72.3.458

[14] Islam M, Doyle MP, Phatak SC, Millner P, Jiang XP (2004) Persistence of enterohemorrhagic Escherichia coli O157:H7 in soil and on leaf lettuce and parsley grown in fields treated with contaminated manure composts or irrigation water. J Food Protect 67: 1365-1370. 10.4315/0362-028x-67.7.1365 15270487

[15] Ercolani GL (1979) Differential survival of *Salmonella typhi*, *Escherichia coli*, and *Enterobacter aerogenes* on lettuce in the field. Zentralbl Bakteriol Naturwiss 134: 402-411. PubMed: 396737. 396737

[16] Moyne AL, Sudarshana MR, Blessington T, Koike ST, Cahn MD et al. (2011) Fate of *Escherichia coli* O157:H7 in field-inoculated lettuce. Food Microbiol 28: 1417-1425. doi:10.1016/j.fm.2011.02.001. PubMed: 21925023. 21925023 10.1016/j.fm.2011.02.001

[17] Tilden J, Young W, McNamara AM, Custer C, Boesel B et al. (1996) A new route of transmission for *Escherichia coli*: Infection from dry fermented salami. Am J Public Health 86: 1142-1145. doi:10.2105/AJPH.86.8_Pt_1.1142. PubMed: 8712275. 8712275 10.2105/ajph.86.8_pt_1.1142 PMC1380627

[18] Griffin PM, Tauxe RV (1991) The epidemiology of infections caused by *Escherichia coli* O157:H7, other enterohemorrhagic *E. coli*, and the associated hemolytic uremic syndrome. Epidemiol Rev 13: 60-98. PubMed: 1765120. 1765120 10.1093/oxfordjournals.epirev.a036079

[19] Choi S, Bang J, Kim H, Beuchat LR, Ryu JH (2011) Survival and colonization of *Escherichia coli* O157:H7 on spinach leaves as affected by inoculum level and carrier, temperature and relative humidity. J Appl Microbiol 111: 1465-1472. doi:10.1111/j.1365-2672.2011.05175.x. PubMed: 21988171. 21988171 10.1111/j.1365-2672.2011.05175.x

[20] Lopez-Velasco G, Davis M, Boyer RR, Williams RC, Ponder MA (2010) Alterations of the phylloepiphytic bacterial community associated with interactions of *Escherichia coli* O157:H7 during storage of packaged spinach at refrigeration temperatures. Food Microbiol 27: 476-486. doi:10.1016/j.fm.2009.12.010. PubMed: 20417396. 20417396 10.1016/j.fm.2009.12.010

[21] Heaton JC, Jones K (2008) Microbial contamination of fruit and vegetables and the behaviour of enteropathogens in the phyllosphere: a review. J Appl Microbiol 104: 613-626. doi:10.1111/j.1365-2672.2007.03587.x. PubMed: 17927745. 17927745 10.1111/j.1365-2672.2007.03587.x

[22] Cooley MB, Chao D, Mandrell RE (2006). Escherichia Coli O157:H7 survival and growth on lettuce is altered by the presence of epiphytic bacteria. J Food Prot 69: 2329-2335. 17066909 10.4315/0362-028x-69.10.2329

[23] Wilson M, Lindow SE (1994) Coexistence among epiphytic bacterial-populations mediated through nutritional resource partitioning. Appl Envion Microbiol 60: 4468-4477. 10.1128/aem.60.12.4468-4477.1994 PMC202007 16349462

[24] Knief C, Ramette A, Frances L, Alonso-Blanco C, Vorholt JA (2010) Site and plant species are important determinants of the *Methylobacterium* community composition in the plant phyllosphere. ISME J 4: 719-728. doi:10.1038/ismej.2010.9. PubMed: 20164863. 20164863 10.1038/ismej.2010.9

[25] Lambais MR, Crowley DE, Cury JC, Büll RC, Rodrigues RR (2006) Bacterial diversity in tree canopies of the Atlantic forest. Science 312: 1917-1917. doi:10.1126/science.1124696. PubMed: 16809531. 16809531 10.1126/science.1124696

[26] De Costa DM, Rathnayake RMPS, De Costa WAJM, Kumari WMD, Dissanayake DMN (2006) Variation of phyllosphere microflora of different rice varieties in Sri Lanka and its relationship to leaf anatomical and physiological characters. J Agron Crop Sci 192: 209-220. doi:10.1111/j.1439-037X.2006.00207.x.

[27] Pedersen JC (1992) Natamycin as a fungicide in agar media. Appl Environ Microbiol 58: 1064-1066. PubMed: 16348667. 16348667 10.1128/aem.58.3.1064-1066.1992 PMC195383

[28] Sakai M, Matsuka A, Komura T, Kanazawa S (2004) Application of a new PCR primer for terminal restriction fragment length polymorphism analysis of the bacterial communities in plant roots. J Microbiol Methods 59: 81-89. doi:10.1016/j.mimet.2004.06.005. PubMed: 15325755. 15325755 10.1016/j.mimet.2004.06.005

[29] Chelius MK, Triplett EW (2001) The diversity of archaea and bacteria in association with the roots of *Zea mays* L. Microb Ecol 41: 252-263. PubMed: 11391463. 11391463 10.1007/s002480000087

[30] Lane D (1991) 16S/23S rRNA sequencing. Stackebrandt E GM, editor. New York: John Wiley & Sons . Nucleic acid techniques in bacterial systematics. pp. 115-147

[31] Rastogi G, Sbodio A, Tech JJ, Suslow TV, Coaker GL et al. (2012) Leaf microbiota in an agroecosystem: spatiotemporal variation in bacterial community composition on field-grown lettuce. ISME J 10: 1812-1822. 10.1038/ismej.2012.32 PMC3446804 22534606

[32] Caporaso JG, Kuczynski J, Stombaugh J, Bittinger K, Bushman FD et al. (2010) QIIME allows analysis of high-throughput community sequencing data. Nat Methods 7: 335-336. doi:10.1038/nmeth.f.303. PubMed: 20383131. 20383131 10.1038/nmeth.f.303 PMC3156573

[33] Li W, Godzik A (2006) Cd-hit: a fast program for clustering and comparing large sets of protein or nucleotide sequences. Bioinformatics 22: 1658-1659. doi:10.1093/bioinformatics/btl158. PubMed: 16731699. 16731699 10.1093/bioinformatics/btl158

[34] Caporaso JG, Bittinger K, Bushman FD, DeSantis TZ, Andersen GL et al. (2010) PyNAST: a flexible tool for aligning sequences to a template alignment. Bioinformatics 26: 266-267. doi:10.1093/bioinformatics/btp636. PubMed: 19914921. 19914921 10.1093/bioinformatics/btp636 PMC2804299

[35] Price MN, Dehal PS, Arkin AP (2010) FastTree 2--approximately maximum-likelihood trees for large alignments. PLOS ONE 5(3): e9490. doi:10.1371/journal.pone.0009490. PubMed: 20224823. 20224823 10.1371/journal.pone.0009490 PMC2835736

[36] Haas BJ, Gevers D, Earl AM, Feldgarden M, Ward DV et al. (2011) Chimeric 16S rRNA sequence formation and detection in Sanger and 454-pyrosequenced PCR amplicons. Genome Res 21: 494-504. doi:10.1101/gr.112730.110. PubMed: 21212162. 21212162 10.1101/gr.112730.110 PMC3044863

[37] Cole JR, Wang Q, Cardenas E, Fish J, Chai B et al. (2009) The Ribosomal Database Project: improved alignments and new tools for rRNA analysis. Nucleic Acids Res 37: 141-145. doi:10.1093/nar/gkp353. PubMed: 19004872. 10.1093/nar/gkn879 PMC2686447 19004872

[38] Faith DP (1992) Conservation evaluation and phylogenetic diversity. Biol Conserv 61: 1-10. doi:10.1016/0006-3207(92)91201-3.

[39] Lozupone C, Hamady M, Knight R (2006) UniFrac - An online tool for comparing microbial community diversity in a phylogenetic context. BMC Bioinformatics 7. 10.1186/1471-2105-7-371 PMC1564154 16893466

[40] Ta H (1999) BioEdit: a user-friendly biological sequence alignment editor and analysis program for Windows 95/98/NT. Nucl Acids Symp Ser 41: 95-98.

[41] Rastogi G, Tech JJ, Coaker GL, Leveau JH (2010) A PCR-based toolbox for the culture-independent quantification of total bacterial abundances in plant environments. J Microbiol Methods 83: 127-132. doi:10.1016/j.mimet.2010.08.006. PubMed: 20816905. 20816905 10.1016/j.mimet.2010.08.006

[42] Andersson AF, Lindberg M, Jakobsson H, Bäckhed F, Nyrén P et al. (2008) Comparative analysis of human gut microbiota by barcoded pyrosequencing. PLOS ONE 3(7): e2836. doi:10.1371/journal.pone.0002836. PubMed: 18665274. 18665274 10.1371/journal.pone.0002836 PMC2475661

[43] Turnbaugh PJ, Hamady M, Yatsunenko T, Cantarel BL, Duncan A et al. (2009) A core gut microbiome in obese and lean twins. Nature 457: 480-487. doi:10.1038/nature07540. PubMed: 19043404. 19043404 10.1038/nature07540 PMC2677729

[44] Sogin ML, Morrison HG, Huber JA, Mark Welch D, Huse SM et al. (2006) Microbial diversity in the deep sea and the underexplored "rare biosphere" Proc Natl Acad Sci U S A 103: 12115-12120. doi:10.1073/pnas.0605127103. PubMed: 16880384. 16880384 10.1073/pnas.0605127103 PMC1524930

[45] Roesch LF, Fulthorpe RR, Riva A, Casella G, Hadwin AK et al. (2007) Pyrosequencing enumerates and contrasts soil microbial diversity. ISME J 1: 283-290. PubMed: 18043639. 18043639 10.1038/ismej.2007.53 PMC2970868

[46] Huber JA, Mark Welch DB, Morrison HG, Huse SM, Neal PR et al. (2007) Microbial population structures in the deep marine biosphere. Science 318: 97-100. doi:10.1126/science.1146689. PubMed: 17916733. 17916733 10.1126/science.1146689

[47] Lopez-Velasco G, Welbaum GE, Boyer RR, Mane SP, Ponder MA (2011) Changes in spinach phylloepiphytic bacteria communities following minimal processing and refrigerated storage described using pyrosequencing of 16S rRNA amplicons. J Appl Microbiol 110: 1203-1214. doi:10.1111/j.1365-2672.2011.04969.x. PubMed: 21371219. 21371219 10.1111/j.1365-2672.2011.04969.x

[48] Redford AJ, Bowers RM, Knight R, Linhart Y, Fierer N (2010) The ecology of the phyllosphere: geographic and phylogenetic variability in the distribution of bacteria on tree leaves. Environ Microbiol 12: 2885-2893. doi:10.1111/j.1462-2920.2010.02258.x. PubMed: 20545741. 20545741 10.1111/j.1462-2920.2010.02258.x PMC3156554

[49] Reisberg EE, Hildebrandt U, Riederer M, Hentschel U (2011) Phyllosphere bacterial communities of trichome-bearing and trichomeless *Arabidopsis thaliana* leaves. Antonie Van Leeuwenhoek 101(3): 551-560. PubMed: 22080429. 22080429 10.1007/s10482-011-9669-8

[50] Whipps JM, Hand P, Pink D, Bending GD (2008) Phyllosphere microbiology with special reference to diversity and plant genotype. J Appl Microbiol 105: 1744-1755. doi:10.1111/j.1365-2672.2008.03906.x. PubMed: 19120625. 19120625 10.1111/j.1365-2672.2008.03906.x

[51] Zwielehner J, Handschur M, Michaelsen A, Irez S, Demel M et al. (2008) DGGE and real-time PCR analysis of lactic acid bacteria in bacterial communities of the phyllosphere of lettuce. Mol Nutr Food Res 52: 614-623. doi:10.1002/mnfr.200700158. PubMed: 18398868. 18398868 10.1002/mnfr.200700158

[52] Hunter PJ, Hand P, Pink D, Whipps JM, Bending GD (2010) Both leaf properties and microbe-microbe interactions influence within-species variation in bacterial population diversity and structure in the lettuce (*Lactuca* species) phyllosphere. Appl Environ Microbiol 76: 8117-8125. doi:10.1128/AEM.01321-10. PubMed: 20952648. 20952648 10.1128/AEM.01321-10 PMC3008232

[53] Ó Cuív P, Aguirre de Cárcer D, Jones M, Klaassens ES, Worthley DL et al. (2011) The effects from DNA extraction methods on the evaluation of microbial diversity associated with human colonic tissue. Microb Ecol 61: 353-362. doi:10.1007/s00248-010-9771-x. PubMed: 21153634. 21153634 10.1007/s00248-010-9771-x

[54] Smith B, Li N, Andersen AS, Slotved HC, Krogfelt KA (2011) Optimising bacterial DNA extraction from faecal samples: Comparison of three methods. Open Microbiol J 5: 14-17. doi:10.2174/1874285801105010014. PubMed: 21643498. 21643498 10.2174/1874285801105010014 PMC3106334

[55] Wu GD, Lewis JD, Hoffmann C, Chen YY, Knight R et al. (2010) Sampling and pyrosequencing methods for characterizing bacterial communities in the human gut using 16S sequence tags. BMC Microbiol 10: 206. doi:10.1186/1471-2180-10-206. PubMed: 20673359. 20673359 10.1186/1471-2180-10-206 PMC2921404

[56] Biesbroek G, Sanders EA, Roeselers G, Wang X, Caspers MP et al. (2012) Deep sequencing analyses of low density microbial communities: working at the boundary of accurate microbiota detection. PLOS ONE 7(3): e32942. doi:10.1371/journal.pone.0032942. PubMed: 22412957. 22412957 10.1371/journal.pone.0032942 PMC3295791

[57] Kunin V, Engelbrektson A, Ochman H, Hugenholtz P (2010) Wrinkles in the rare biosphere: pyrosequencing errors can lead to artificial inflation of diversity estimates. Environ Microbiol 12: 118-123. doi:10.1111/j.1462-2920.2009.02051.x. PubMed: 19725865. 19725865 10.1111/j.1462-2920.2009.02051.x

[58] Jackson CR, Denney WC (2011) Annual and seasonal variation in the phyllosphere bacterial community associated with leaves of the southern Magnolia (*Magnolia grandiflora*). Microb Ecol 61: 113-122. doi:10.1007/s00248-010-9742-2. PubMed: 20809288. 20809288 10.1007/s00248-010-9742-2

[59] Redford AJ, Fierer N (2009) Bacterial succession on the leaf surface: A novel system for studying successional dynamics. Microb Ecol 58: 189-198. doi:10.1007/s00248-009-9495-y. PubMed: 19221834. 19221834 10.1007/s00248-009-9495-y

[60] Bell CW, Acosta-Martinez V, McIntyre NE, Cox S, Tissue DT et al. (2009) Linking microbial community structure and function to seasonal differences in soil moisture and temperature in a Chihuahuan desert grassland. Microb Ecol 58: 827-842. doi:10.1007/s00248-009-9529-5. PubMed: 19466479. 19466479 10.1007/s00248-009-9529-5

[61] Lipson DA, Schmidt SK (2004) Seasonal changes in an alpine soil bacterial community in the Colorado Rocky Mountains. Appl Envion Microbiol 70: 2867-2879. doi:10.1128/AEM.70.5.2867-2879.2004. 10.1128/AEM.70.5.2867-2879.2004 PMC404381 15128545

[62] Bowers RM, McCubbin IB, Hallar AG, Fierer N (2012) Seasonal variability in airborne bacterial communities at a high-elevation site. Atmos Environ 50: 41-49. doi:10.1016/j.atmosenv.2012.01.005.

[63] Finkel OM, Burch AY, Lindow SE, Post AF, Belkin S (2011) Geographical location determines the population structure in phyllosphere microbial communities of a salt-excreting desert tree. Appl Envion Microbiol 77: 7647-7655. doi:10.1128/AEM.05565-11. 10.1128/AEM.05565-11 PMC3209174 21926212

[64] Koopman MM, Fuselier DM, Hird S, Carstens BC (2010) The carnivorous pale pitcher plant harbors diverse, distinct, and time-dependent bacterial communities. Appl Environ Microbiol 76: 1851-1860. doi:10.1128/AEM.02440-09. PubMed: 20097807. 20097807 10.1128/AEM.02440-09 PMC2838028

[65] Hirano SS, Upper CD (1989) Diel variation in population-size and ice nucleation activity of *Pseudomonas syringae* on snap bean leaflets. Appl Envion Microbiol 55: 623-630. 10.1128/aem.55.3.623-630.1989 PMC184170 16347871

[66] Zhang B, Bai Z, Hoefel D, Tang L, Yang Z et al. (2008) Assessing the impact of the biological control agent *Bacillus thuringiensis* on the indigenous microbial community within the pepper plant phyllosphere. FEMS Microbiol Lett 284: 102-108. doi:10.1111/j.1574-6968.2008.01178.x. PubMed: 18462395. 18462395 10.1111/j.1574-6968.2008.01178.x

[67] Lopez-Velasco G, Tydings HA, Boyer RR, Falkinham JO, Ponder MA (2012) Characterization of interactions between *Escherichia coli* O157:H7 with epiphytic bacteria *in vitro* and on spinach leaf surfaces. Int J Food Microbiol 153: 351-357. doi:10.1016/j.ijfoodmicro.2011.11.026. PubMed: 22177225. 22177225 10.1016/j.ijfoodmicro.2011.11.026

[68] Larsen P, Hamada Y, Gilbert J (2012) Modeling microbial communities: Current, developing, and future technologies for predicting microbial community interaction. J Biotechnol 160: 17-24. doi:10.1016/j.jbiotec.2012.03.009. PubMed: 22465599. 22465599 10.1016/j.jbiotec.2012.03.009

