# Supplementary figures and images for: Season, Irrigation, Leaf Age, and Escherichia coli Inoculation Influence the Bacterial Diversity in the Lettuce Phyllosphere
Source: PLoS One. 2013 Jul 2;8(7):e68642. doi: 10.1371/journal.pone.0068642 (PMC3699665; doi:10.1371/journal.pone.0068642)

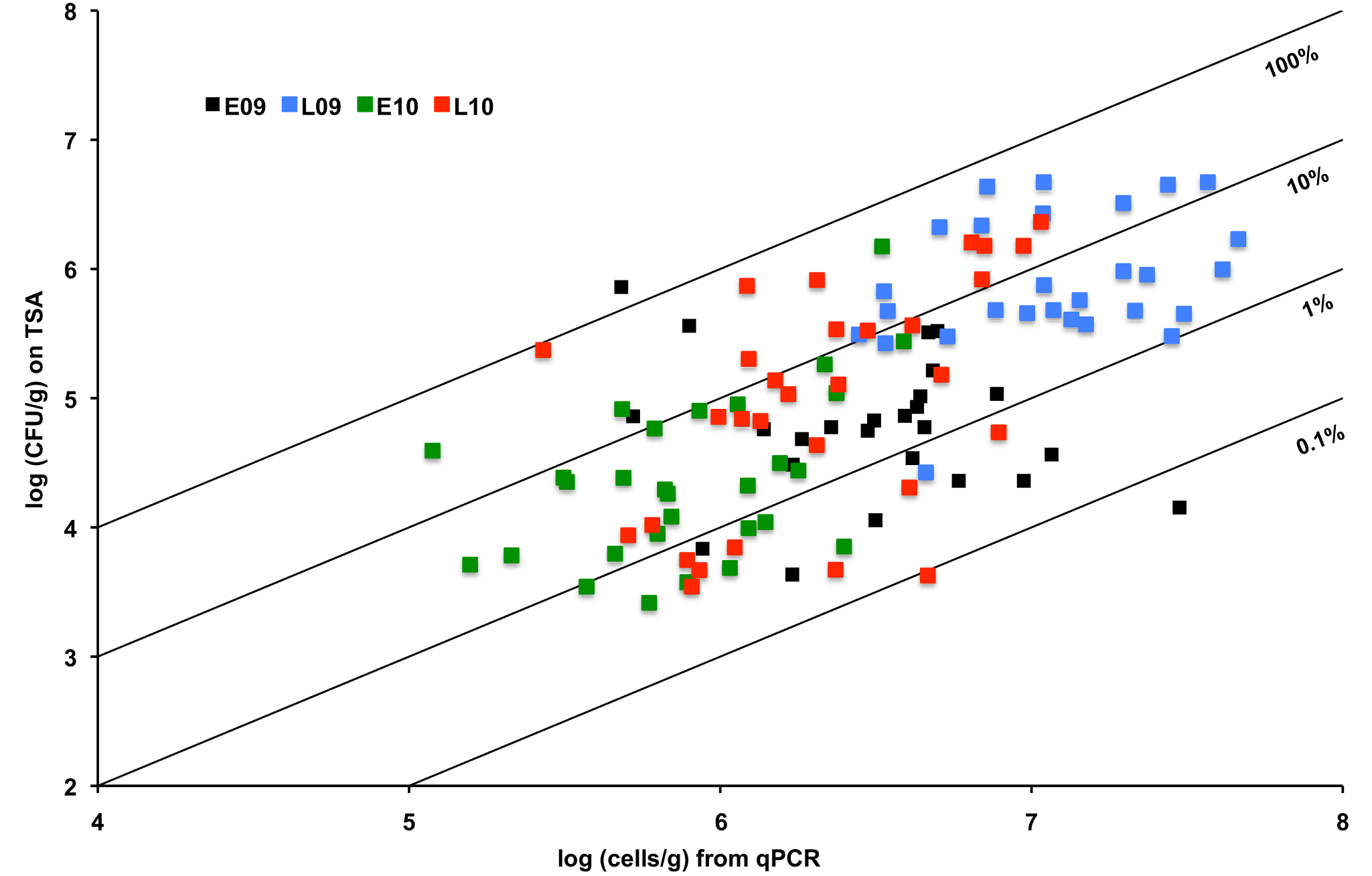

Supplement: Figure S1 — The estimated number of bacterial cells per gram lettuce determined by quantitative real-time PCR targeting the bacterial 16S rRNA genes compared with colony forming units enumerated on TSA. The lines show the percent of total bacteria detected by TSA compared with real-time PCR. Each point represents an individual plant collected at the 7, 14, and 21 dpi time points from each field trial; n=115. (TIF) [file pone.0068642.s001.tif]

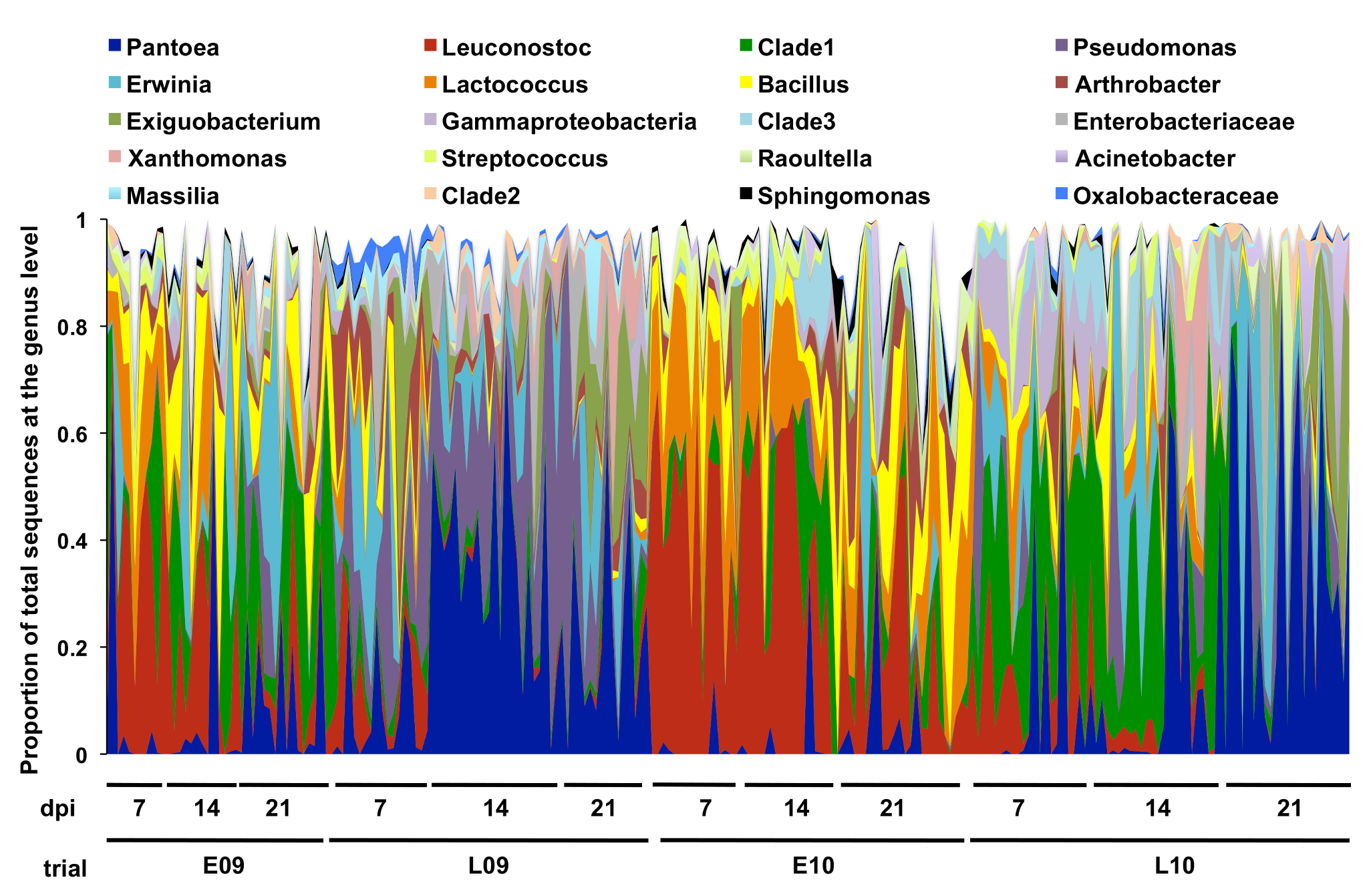

Supplement: Figure S4 — The top 20 most abundant taxonomic groups at the genus level are listed. The relative abundance for each of the 20 groups is shown for each plant, listed from 7 dpi (left) to 21 dpi (right) for the four field trials. When sequences were unable to be classified to a genus, the next most specific designation is shown (i.e. Family or Class level). (TIF) [file pone.0068642.s004.tif]

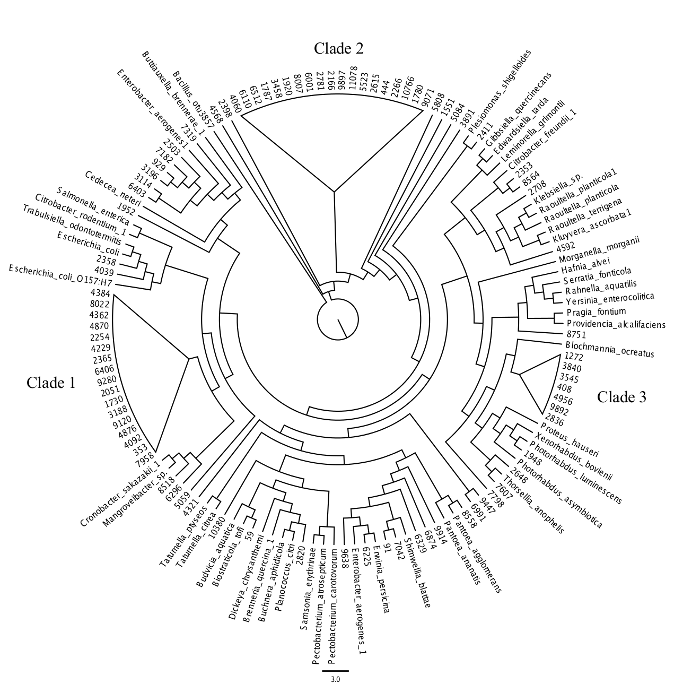

Supplement: Figure S6 — The similarity of 16S rRNA sequences from OTU’s classified as belonging to unknown genera of Enterobacteriaceae (numbers) and representative Enterobacteriaceae genera (Genus_species) is investigated here. Those OTU’s that grouped together and not closely related to known genera were designated as belonging to one of three Clades. Individual branches for OTUs within a clade were removed to improve visualization. The average of sequence lengths for the Enterobacteriaceae unknowns (417 bp) was not significantly different from the average read length of all OTUs (431 bp) in the data set (P > 0.15, by Student’s T-test). An OTU classified as Bacillus was used as an outgroup. (TIF) [file pone.0068642.s006.tif]

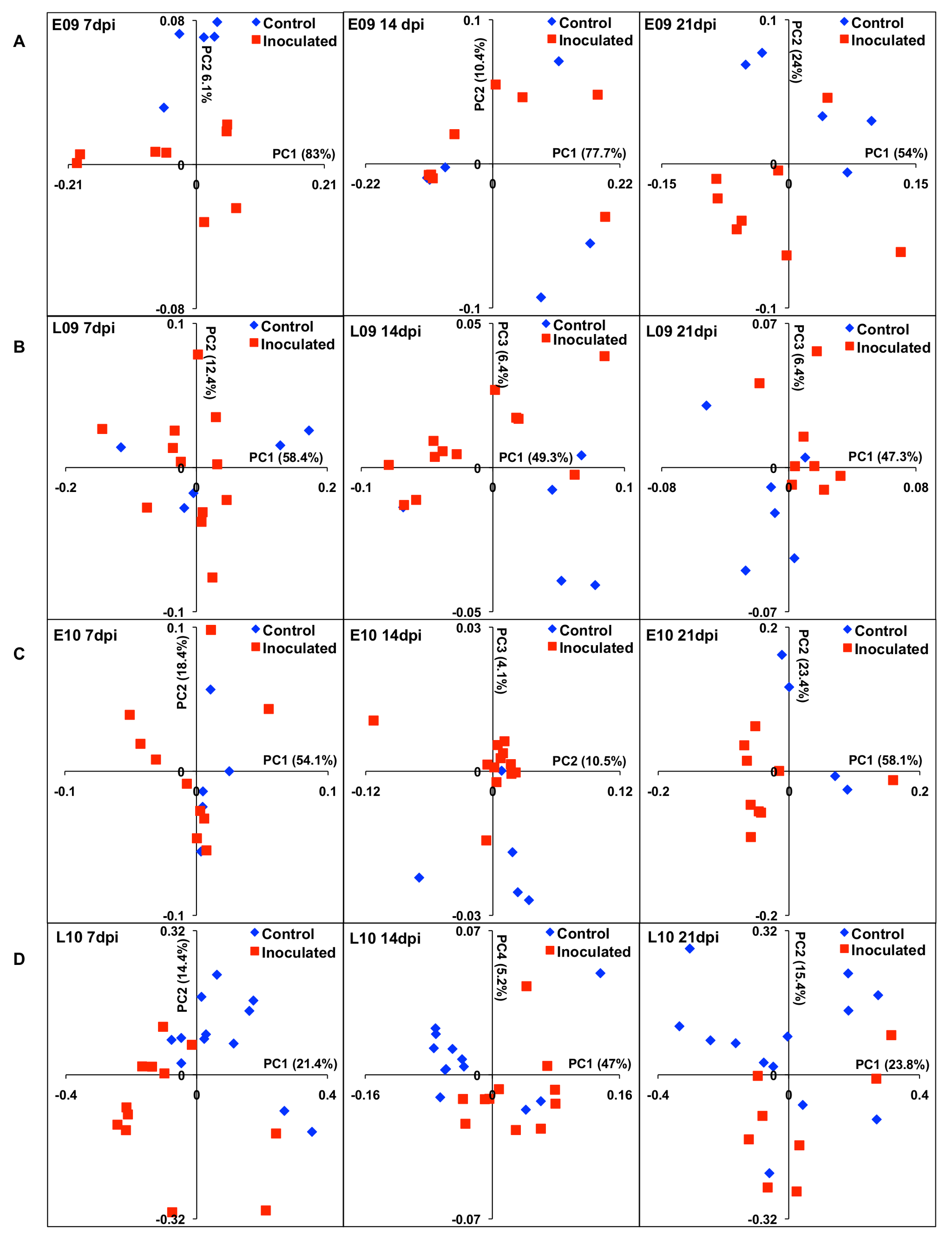

Supplement: Figure S7 — Differences between inoculated and control plants are highlighted at each timepoint in all four trials. Unweighted UniFrac community distance data was used to make the E09 14 dpi drip-irrigated PCoA, the remaining analyses used the weighted UniFrac data. All points represent a single microbiota isolated from lettuce. (A) E09; (B) L09; (C) E10; (D) L10. (TIF) [file pone.0068642.s007.tif]

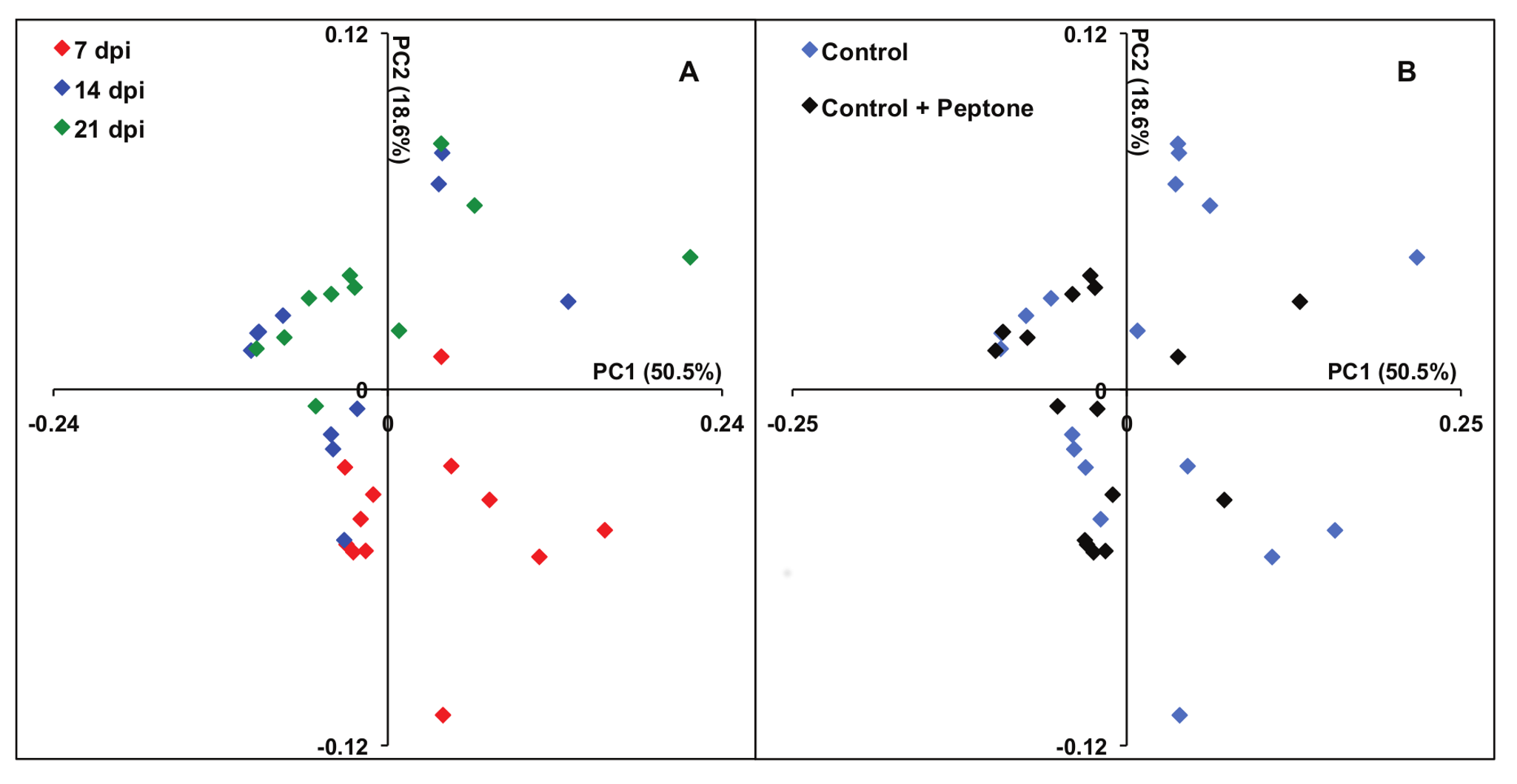

Supplement: Figure S8 — PCoA plots of weighted UniFrac community distances of control plants in the L10 trial. Plant samples are colored according to days post inoculation (dpi) (A) and control group type (unsprayed controls (control) vs. peptone sprayed controls (control + peptone) (B). (TIF) [file pone.0068642.s008.tif]
