# Supplementary material for: Season, Irrigation, Leaf Age, and Escherichia coli Inoculation Influence the Bacterial Diversity in the Lettuce Phyllosphere
Source: PLoS One. 2013 Jul 2;8(7):e68642. doi: 10.1371/journal.pone.0068642 (PMC3699665; doi:10.1371/journal.pone.0068642)
Supplement: Table S1 — Temperature and humidity measurements in the field. (DOC) [file pone.0068642.s009.doc]

| **Table S1.** Temperature and humidity measurements in the field | | | | |
| --- | --- | --- | --- | --- |
|  | **Average T (°C)** | **Min / Max T (°C)** | **Average RH** | **Min / Max RH** |
| **E09** | 16.19 ± 3.37 | 7.87 / 30.80 | 75.9 ± 11.3 | 35.3 / 95.2 |
| **L09** | 13.99 ± 4.98 | 2.58 / 30.14 | 76.1 ± 16.7 | 24.3 / 96.5 |
| **E10** | 14.75 ± 3.85 | 8.59 / 31.10 | 78.6 ± 12.6 | 25.9 / 96.5 |
| **L10** | 16.70 ± 4.96 | 7.42 / 37.54 | 77.9 ± 16.0 | 21.1 / 97.6 |
| Temperature (T); Relative Humidity (RH)  Average readings are from measurements taken 24 hours/day over a minimum of 15 min intervals beginning at the time of *E. coli* O157:H7 inoculation for 28 days. | | | | |
