# Supplementary material for: Season, Irrigation, Leaf Age, and Escherichia coli Inoculation Influence the Bacterial Diversity in the Lettuce Phyllosphere
Source: PLoS One. 2013 Jul 2;8(7):e68642. doi: 10.1371/journal.pone.0068642 (PMC3699665; doi:10.1371/journal.pone.0068642)
Supplement: Table S2 — Percentage of inoculated plants with persistent E. coli O157:H7 populations. (DOC) [file pone.0068642.s010.doc]

| **Table S2.** Percentage of inoculated plants with persistent *E. coli* O157:H7 populations | | | | | |
| --- | --- | --- | --- | --- | --- |
| **Trial** | **2dpi** | **7dpi** | **14dpi** | **21dpi** | **28dpi** |
| June 2009 | 79.2 | N/A* | 16.7 | 12.5 | 0 |
| October 2009 | 87.5 | 62.5 | 37.5 | 8.3 | 0 |
| June 2010 | 91.7 | 58.3 | 25.0 | 25.0 | 12.5 |
| August 2010 | 58.3 | 54.2 | 12.5 | 8.3 | 0 |
| * Enrichment results not determined. n=24. | | | | | |
